# Alisol F 24-acetate attenuated metabolic dysfunction-associated steatohepatitis by targeting the KEAP1/NRF2-mediated macrophage pyroptosis

**DOI:** 10.1186/s13020-025-01322-8

**Published:** 2026-01-13

**Authors:** Zhiwu Dong, Keliang Huang, Weiyi Wu, Lianxiang Xing, Ying Zhang, Xin Zhang, Wenwei Yang, Kewen Zhao

**Affiliations:** 1 Department of Laboratory Medicine, Shanghai Second People’s Hospital, No. 58, East Puyu Road, Huangpu District, Shanghai, 200011 China; 2 Key Laboratory of Cell Differentiation and Apoptosis of the National Ministry of Education, Shanghai Frontiers Science Center of Cellular Homeostasis and Human Diseases, Department of Pathophysiology, Shanghai Jiao Tong University School of Medicine, Shanghai, 200025 China

**Keywords:** Metabolic dysfunction-associated steatohepatitis, Alisol F 24-acetate, Pyroptosis, Reactive oxygen species, KEAP1/NRF2 signaling pathway

## Abstract

**Background:**

Metabolic dysfunction-associated steatohepatitis (MASH) is a severe progressive subtype of metabolic-related fatty liver disease that is defined by hepatic steatosis, hepatocyte damage, inflammation, and fibrosis. Alisol F 24-acetate (ALI), a triterpene derived from Rhizoma Alismatis, has anti-inflammatory and antioxidant properties. This study aimed to evaluate the therapeutic effects of ALI in a mouse model of MASH, RAW264.7 cells, and bone marrow-derived macrophages (BMDMs).

**Methods:**

Levels of serum biochemicals, pathological changes in the liver, pyroptosis, and expression of the Kelch-like ECH-associated protein 1(KEAP1)/Nuclear factor E2-related factor 2 (NRF2) pathway were assessed in mice fed a methionine–choline-deficient (MCD) diet with different doses of ALI. Lipopolysaccharide (LPS)-stimulated RAW264.7 cells and BMDMs were used to ascertain the potential mechanisms of ALI on macrophage polarization.

**Results:**

We found that ALI supplementation in MCD-fed mice decreased liver pathology, lipid accumulation, inflammation, and fibrosis. Moreover, ALI could attenuate M1 polarization, promote M2 polarization, suppress pyroptosis, and reduce oxidative stress levels via the KEAP1/NRF2 signaling pathway in tissue samples. ALI also suppressed LPS-induced RAW264.7 cells and BMDMs pyroptosis by inhibiting NLRP3 activation and reducing the level of reactive oxygen species. Molecular docking results suggested that ALI could bind with KEAP1. Overexpressing *Keap1* weakened the effects of ALI on pyroptosis and affirmed a role associated with KEAP1/NRF2 pathways in macrophage.

**Conclusion:**

Our findings suggest that ALI suppressed macrophage pyroptosis by targeting KEAP1/NRF2 interactions, providing reliable data on the protective mechanism of natural antioxidants against MASH.

**Supplementary Information:**

The online version contains supplementary material available at 10.1186/s13020-025-01322-8.

## Background

Metabolic dysfunction-associated steatohepatitis (MASH), previously known as non-alcoholic steatohepatitis (NASH), is a progressive and severe form of metabolic-related fatty liver disease, characterized by hepatic steatosis, hepatocyte injury, inflammation, and fibrosis [1]. This progressive liver disease is closely linked to metabolic disorders such as obesity and type 2 diabetes [1, 2]. MASH is estimated to affect 2–6% of the global population, with a 50% increase in its prevalence projected by 2030, driven by rising rates of obesity, diabetes, and metabolic syndrome [2–4]. Despite its growing impact, MASH remains underdiagnosed due to limited awareness, non-specific symptoms, and the lack of widely available diagnostic tools.

The current therapeutic options for MASH are largely restricted to lifestyle modifications, such as diet and exercise, whereas pharmacological intervention is still under development [1]. This emphasizes the urgent need for effective molecular methods to address the burden of MASH. The intricate mechanisms underpinning MASH pathology involve a combination of lipid metabolism dysfunction, oxidative stress, immune responses, and cellular death, including pyroptosis, a highly inflammatory form of programmed cell death [5, 6]. Targeting these pathways may offer a potential avenue for developing effective interventions against disease progression.

Macrophages, crucial components of innate immunity and inflammation, play pivotal roles in liver function. Liver-resident Kupffer cells and monocyte-derived macrophages (MDMs) are key macrophage types involved in regulating inflammation and fibrogenesis [7]. M1 to M2 macrophage polarization plays a pivotal role in modulating inflammation and oxidative stress in various diseases, including MASH [8]. M1 macrophages can release inflammatory chemokines and cytokines to exert pro-inflammatory effects [9]. Conversely, M2 macrophages are involved in hepatocyte repair and immune tolerance to inhibit inflammatory responses [10]. Notably, many of these macrophage-derived cytokines activate hepatic stellate cells (HSCs), the primary effector cells in liver fibrosis [11, 12]. Recently, pyroptosis, a form of inflammatory cell death, has attracted more attention in the development of MASH [13]. It is triggered by Caspase-1 in response to signals from NOD-like receptor protein 3 (NLRP3) inflammasomes. Upon activation, pro-Caspase-1 is cleaved into p20 and p10 subunits, which assemble into the active Caspase-1 enzyme. It then interacts with and cleaves Gasdermin D (GSDMD), a pore-forming protein [14]. Several studies have demonstrated that NLRP3 is highly associated with MASH and liver fibrosis, and that the high expression of NLRP3 is primarily derived from MDMs [15, 16]. Transitioning macrophages from the M1 to M2 state can suppress pyroptosis by downregulating the NLRP3 inflammasome and related pathways [17]. These findings suggest that inhibiting macrophage pyroptosis may represent a novel strategy for preventing MASH.

The KEAP1/NRF2 signaling pathway is central to reducing oxidative stress [18]. NRF2, when activated, translocates to the nucleus and upregulates the expression of antioxidant response elements, including heme oxygenase-1, catalase, and glutathione peroxidase [19]. These enzymes neutralize reactive oxygen species (ROS), thereby mitigating oxidative damage [20]. M2 polarization enhances NRF2 activity, creating a feedback loop that further suppresses oxidative stress and inflammation [21]. In the context of MASH, this mechanism is particularly relevant as oxidative stress and inflammation are key drivers of disease progression [22]. The promotion of M1 to M2 polarization to activate the KEAP1/NRF2 pathway could potentially alleviate the pathological features of MASH.

Natural compounds derived from traditional medicinal sources have attracted substantial interest because of their potential therapeutic properties. Several are known to ameliorate oxidative stress and promote macrophage polarization [23]. Alisol F 24-acetate (ALI), a triterpene isolated from Alismatis Rhizoma, has demonstrated various pharmacological effects, including anti-inflammatory and antioxidant activities [24]. However, whether it can ameliorate MASH-associated liver damage and inflammation is unclear.

This study aims to investigate the pharmaceutical effects of ALI in a methionine–choline-deficient (MCD) diet-induced mouse model of MASH and lipopolysaccharide (LPS)-stimulated murine-derived RAW264.7 cells and bone marrow-derived macrophages (BMDMs). By assessing changes in serum biochemistry, liver pathology, pyroptosis, macrophage polarization, and oxidative stress, this study seeks to uncover the therapeutic potential of ALI, as well as underlying mechanisms associated with the KEAP1/NRF2 signaling pathway. We also evaluate the molecular docking of ALI with key molecular targets to determine whether it can be used as a natural antioxidant and anti-inflammatory therapeutic for MASH.

## Methods

### Chemicals and reagents

The chemicals and reagents used in this study include Alisol F 24-acetate (Cas: 443683-76-9, TargetMol, Shanghai, China), lipopolysaccharide (LPS, SMB00610, Sigma‒Aldrich, St. Louis, MO, USA), and adenosine triphosphate (ATP, A2383, Sigma‒Aldrich, St. Louis, MO, USA). Dulbecco’s modified Eagle’s medium (DMEM), fetal calf serum (FCS), and penicillin/streptomycin (ExCell Bio, Shanghai, China). Assay kits for alanine aminotransferase (ALT), aspartate aminotransferase (AST), triglyceride (TG), cholesterol (TC), superoxide dismutase (SOD), malondialdehyde (MDA), and glutathione (GSH) from Nanjing Jiancheng Institute of Biotechnology (Nanjing, China).

### Construction of lentiviral vectors

Mouse *Keap1* (GenBank accession number AB020063.1) full-length coding sequences were synthesized and subcloned into a GV492 vector (GeneChem, Shanghai, China). The resulting mouse GV492-*Keap1* constructs were sequenced to confirm correct insertion. The vectors were co-transfected into 293 T cells using Lipo3000 (Thermo Fisher Scientific, Waltham, MA, USA) according to the manufacturer’s instructions. After 72 h, lentiviruses were harvested and stored at − 80 °C.

### Animals and treatments

Fifty-five male C57BL/6 mice, 6–7 weeks of age (20–25 g), were purchased from Beijing Vital River Experimental Animal Technology Co. Ltd. (Beijing, China) and kept in a pathogen-free environment at a controlled temperature (24 ± 2 ℃) and humidity (60 ± 5%) with a 12 h light/12 h dark cycle for 7 days. Mice were randomly assigned to control or treatment groups. The control mice (CON and CON + H-ALI) received a normal chow diet throughout the experiment. The MASH mice received a methionine–choline-deficient (MCD) diet (Research Diets, Inc., New Brunswick, NJ, USA). After 2 weeks of feeding a normal chow or MCD diet, various doses of ALI (10, 20, or 30 mg/kg) were administered to the ALI treatment groups by daily oral gavage for 8 weeks, while mice in the CON and MASH control groups received an equal volume of saline. The mice treatment groups were as follows: (1) CON, untreated control group receiving normal chow; (2) CON + H-ALI, normal diet for 2 weeks then administered a high dose of ALI (30 mg/kg) for 8 weeks; (3) MASH, MCD diet throughout the experiment; (4) MASH + L-ALI, MCD diet for 2 weeks then MCD diet with low dose of ALI (10 mg/kg) for 8 weeks; (5) MASH + M-ALI, MCD diet for 2 weeks then MCD diet with medium dose of ALI (20 mg/kg) for 8 weeks; (6) MASH + H-ALI, MCD diet for 2 weeks then MCD diet with high dose of ALI (30 mg/kg) for 8 weeks.

For lentiviral interventions, C57BL/6 mice were fed with a normal diet, MCD diet, or MCD diet with ALI treatment. Two groups from the MCD diet with ALI treatment were given a tail vein injection of LV-Ctrl or LV-*Keap1* packaged lentivirus (5 × 10^8^ TU/ml, 100 μL) once every 2 weeks from the beginning of the experiment. All groups were euthanized after 10 weeks. The successful delivery of KEAP1 was confirmed by western blot analysis of the target protein as previously described [25].

Mice were weighed once a week for the 10-week duration of the experiment. Mice were cared for humanely in compliance with established animal laboratory regulations. After 10 weeks, the mice were killed via intraperitoneal injection of sodium pentobarbital (100 mg/kg body weight), and tissues, including blood and liver, were obtained and cryopreserved at − 80 °C.

### Preparation of tissue and serum samples

Mice were killed humanely via i.p. injection of sodium pentobarbital (100 mg/kg body wt). Blood samples were collected and centrifuged at 3000 × g at 4 ℃ for 10 min to obtain serum. Livers were removed and weighed. Part of the liver tissue was fixed in 4% paraformaldehyde and embedded in paraffin for immunohistochemical staining, while the rest was frozen in liquid nitrogen and stored at − 80 ℃ for further analysis.

### Histological and immunohistochemical analysis

Formaldehyde-fixed paraffin-embedded liver tissue samples were sliced into 4-μm sections and mounted onto slides. For immunohistochemical analysis, sections were incubated with primary antibodies against Col1 or F4/80 overnight at 4 °C, followed by secondary antibodies. DAB chromogen solution was added to the sections and counterstained with hematoxylin. Sections were visualized by microscopy. Positive cells were identified by brown-yellow granules in the cytoplasm or nucleus, and the percentage was determined.

### Hematoxylin and eosin (H&E) staining

H&E staining was performed on paraformaldehyde-fixed paraffin-embedded liver tissue samples using an H&E staining kit (abcam, Cambridge, UK). A value for the metabolic dysfunction-associated steatotic liver disease (MASLD) activity score was determined by two independent liver pathologists blinded to the study. Samples were scored according to the MASH Clinical Research Network Scoring System, which measured liver steatosis (based on the number of hepatocytes), liver inflammation (based on the number of inflammatory foci), and stages of fibrosis.

### Oil red O staining

Briefly, fixed samples were washed in water and then rinsed with 60% isopropanol. Samples were stained with freshly prepared oil red O working solution (Sigma-Aldrich, St. Louis, MO, USA) for 15 min. After rinsing in 60% isopropanol, nuclei were counterstained with hematoxylin (Sangon Biotech).

### Immunofluorescence staining

Levels of oxidative stress in liver tissue samples were measured by immunofluorescence with a ROS detection kit (Thermo Scientific, Waltham, MA, USA). Briefly, liver Sections (4 μm) were dewaxed in xylene, hydrated in decreasing concentrations for 30 min, washed with phosphate-buffered saline (PBS), and probed with monoclonal antibodies or isotype controls at 4 °C overnight. After being washed, the sections were incubated with biotinylated goat anti-rabbit or anti-mouse IgG at room temperature for 2 h. Immunostaining was visualized with streptavidin/peroxidase complex and diaminobenzidine, and sections were counterstained with hematoxylin. Slides were visualized under a bright-field microscope at × 40 and × 400 magnification. Immunofluorescence staining images were taken using a ZEISS microscope (LSM880, Germany). Positive cells were quantified using ImageJ software (NIH, USA) and expressed as mean ± SEM in high-powered fields detected by confocal microscopy.

### Cell culture and treatment

The mouse macrophage cell line, RAW264.7, was obtained from the Type Culture Collection of the Chinese Academy of Sciences. RAW264.7 cells were grown in DMEM containing 10% FCS and 1% penicillin/streptomycin (P/S) at 37 °C in an atmosphere of 5% CO_2_.

Bone marrow cells were harvested aseptically by flushing the femur and tibia with ice-cold PBS. Erythrocytes were subsequently removed using a commercial RBC lysis buffer (eBioscience, Carlsbad, CA, USA). The isolated cells were then seeded into six-well plates and maintained in DMEM supplemented with 10% fetal bovine serum (FBS). To induce differentiation into bone marrow-derived macrophages (BMDMs), the cultures were treated with 20 ng/ml macrophage colony-stimulating factor (M-CSF; BioLegend, San Diego, CA, USA, Cat# 576404) for 7 days. Prior to any experimental stimulation, cell viability was assessed via trypan blue exclusion staining (Invitrogen, Carlsbad, CA, USA).

RAW264.7 cells and BMDMs were seeded into 96-well plates and six-well plates until 60–70% confluence and incubated in LPS (250 ng/ml) overnight to induce a pro-inflammatory macrophage model. Subsequently, cells were co-treated with LPS (250 ng/ml) and ALI (0, 5, 10, or 20 μM) for 24 h, followed by CCK-8, qRT-PCR, and western blot analyses.

### Western blot analysis

We used RIPA buffer (Beyotime) to extract proteins from cells. A BCA assay kit (Invitrogen) was used to measure protein concentration. Proteins were separated using 10% SDS-PAGE and transferred to polyvinylidene fluoride membranes (Bio-Rad, Hercules, CA, USA). After blocking with 5% skimmed milk, membranes were incubated with a primary antibody at 4 °C overnight. The antibodies used in the study were CD86 (#19589, Cell Signaling Technology (CST), Danvers, MA, USA, 1:1000); iNOS (ab178945, abcam, 1:1000); CD206 (ab64693, abcam, 1:1000) Arg1 (#93668, CST, 1:1000); NLRP3 (ab270449, abcam, 1:1000) N-GSDMD (#10137, CST, 1:1000); GSDMD (#39754, CST, 1:1000); cleaved Caspase 1 (c-Caspase 1, #89332, CST, 1:1000); Caspase 1 (ab138483, abcam, 1:1000); KEAP1 (ab119403, abcam, 1:1000); NRF2 (#12721, CST, 1:1000); SOD1 (ab308181, abcam, 1:1000); SOD2 (ab68155, abcam, 1:1000); tumor necrosis factor (TNF-α) (ab183218, abcam, 1:1000); interleukin (IL)−1β (ab315084, abcam, 1:1000); p-P65 (#3033, CST, 1:1000); P65 (#8242, CST, 1:1000); and Histone H3 (ab1791, abcam, 1:5000). After washing, the membranes were incubated with horseradish peroxidase-conjugated secondary antibody (CST) for an hour at room temperature. Protein bands were visualized using a western blotting detection system (CST) according to the manufacturer’s instructions.

### Flow cytometry analysis

Livers were collected from mice following the in vivo experiments. The tissues were minced and dissociated in cell culture medium containing 0.01% type IV collagenase and 0.001% DNase I for 45 min at 37 °C. The mixture was subsequently filtered carefully through a 70-μm nylon cell strainer to obtain a single-cell suspension. After centrifugation at 70 × g for 3 min at 4 °C, the supernatants were discarded, and the pellets were resuspended in 3 ml HBSS. After erythrocyte lysis, samples were centrifuged for 5 min at 500 × g, 4 °C, and then washed 2 times. The final concentration was adjusted to 1 × 10^7^ cells/ml. They were then added to F4/80 antibody and CD206 or F4/80 and CD86 (GeneTex, SC, USA) and incubated on ice in the dark for 30 min. The prepared samples were measured and analyzed using a Beckman Cyto FLEX flow cytometer (Beckman Bioscience, USA). M1 and M2 macrophages were F4/80^+^ CD86^+^ and F4/80^+^ CD206^+^, respectively. Their ratio to the number of negative cells was obtained to determine the polarization level of M1 or M2 macrophages.

### Molecular docking

The molecular docking values of the ALI protein were determined by AutoDock software using two-dimensional structures downloaded from the RCSB-PDB Database. After running the docking simulation, the results were visualized using PyMOL.

### Cellular thermal shift assay (CETSA)

To independently confirm the direct interaction between ALI and KEAP1, a cellular thermal shift assay (CETSA) was employed, followed by Western blot analysis. In brief, after a 24-h treatment with ALI, cells were washed twice with ice-cold PBS and lysed using RIPA buffer containing protease and phosphatase inhibitors. The lysates were centrifuged (15,000 × g, 20 min, 4 °C) to obtain the soluble protein fraction. The supernatant was divided equally into six aliquots, each exposed to a distinct temperature (50, 55, 60, 65, 70, or 75 °C) for 3 min. After heating, samples were cooled at 4 °C for an equal duration. Subsequently, the remaining soluble proteins were denatured in 1 × loading buffer at 97 °C for 7 min. The thermal stability of KEAP1 was finally evaluated and quantified via Western blot.

### RT-qPCR analysis

Total RNA was extracted from cells or tissue by Trizol. A Prime Script RT Mix reagent Kit (TakaRa, Dalian, China) was used to reverse transcribe 1 μg of mRNA to cDNA. Quantitative PCR was conducted using a Bio-Rad CFX96 Real-Time PCR Detection System and SYBR Green Supermix (Bio-Rad). The primers are given in Table 1. Amplification conditions were 1 cycle of 95 °C for 2 min followed by 40 cycles of 95 °C for 10 s, 60 °C for 30 s, and 72 °C for 30 s. The relative expression of each gene was normalized to the expression of GAPDH. Relative expression was calculated using the 2^−ΔΔCt^ method.

**Table 1:** PCR primers used in this study

	Forward (5′–3′)	Reverse (5′–3′)
TNF-α	CTGAACTTCGGGGTGATCGG	GGCTTGTCACTCGAATTTTGAGA
IL-6	CTGCAAGAGACTTCCATCCAG	AGTGGTATAGACAGGTCTGTTGG
IL-10	GCTCTTACTGACTGGCATGAG	CGCAGCTCTAGGAGCATGTG
IL-1β	GAAATGCCACCTTTTGACAGTG	TGGATGCTCTCATCAGGACAG
α-Sma	CCCAGACATCAGGGAGTAATGG	TCTATCGGATACTTCAGCGTCA
Tgf-β	CTTCAATACGTCAGACATTCGGG	GTAACGCCAGGAATTGTTGCTA
Col 1	CTGGCGGTTCAGGTCCAAT	TTCCAGGCAATCCACGAGC
GAPDH	TGGCCTTCCGTGTTCCTAC	GAGTTGCTGTTGAAGTCGCA

### Enzyme-linked immunosorbent assay (ELISA)

ELISA kits (Thermo Fisher Scientific) were used to quantify levels of TNF-α, IL-6, IL-10, and IL-1β in liver tissue.

### Cell viability assay

The viability of RAW264.7 cells and BMDMs was assessed using a Cell Counting Kit-8 (CCK8, Dojindo, Kumamoto, Japan). Cells treated with different concentrations of ALI were cultured in DMEM in 96-well plates (1 × 10^4^ cells/well) for 24 h at 37 °C. CCK8 reagent was added to the plates, which were incubated for a further 4 h. Absorbances were detected at 450 nm.

### Transmission electron microscopy

To analyze the cellular ultrastructural alterations, transmission electron microscopy (TEM) was performed on RAW264.7 cells. Cells were seeded at a density of 3 × 10^5^ cells per well and allowed to adhere overnight. Following adhesion, they were incubated for 24 h with LPS (250 ng/mL), either with or without the addition of ALI (20 μM). After treatment, cells were washed with PBS and primarily fixed for 1 h in a solution of 4% paraformaldehyde and 2% glutaraldehyde. Subsequently, a secondary fixation was conducted using 1% osmium tetroxide and 0.5% potassium ferricyanide in cacodylate buffer for an additional hour. The fixed specimens were then embedded in resin, followed by polymerization at 80 °C for 24 h. Ultrathin sections were examined using a Tecnai G2 Spirit Twin microscope (Thermo Fisher Scientific) and a JEM ARM 1300S high-voltage electron microscope (JEOL) for detailed ultrastructural observation.

### Lactate dehydrogenase (LDH) analysis

LPS-stimulated RAW264.7 cells and BMDMs were cultured in 96-well plates with different concentrations of ALI (0, 5, 10, or 20 μM) and ATP (5 mM). After 6 h, the level of LDH was measured in the culture media using an LDH Cytotoxicity Assay Kit (Beyotime, Shanghai, China) following the manufacturer’s instructions.

### Measurement of intracellular oxidative stress levels

Total and mitochondrial ROS levels were detected by using dichlorodi-hydrofluorescein diacetate (DCFH-DA) (Beyotime). Briefly, macrophages were incubated with DCFH-DA at room temperature for 30 min in the dark. Fluorescent intensity was determined by fluorescent microscopy. MDA and GSH levels and SOD activity in cell homogenate and mouse serum were measured according to the manufacturer’s instructions (Beyotime).

### Hoechst/propidium iodide (PI) staining

RAW264.7 cells and BMDMs were stained with PI and counterstained with Hoechst. Cells were then washed with ice-cold PBS and observed under a fluorescent microscope (Olympus, Tokyo, Japan).

### Statistical analysis

Statistical analyses were performed using SPSS 22.0 software (SPSS, Chicago, IL, USA). Graphical representations and image-based analyses were conducted using GraphPad Prism (GraphPad Software Inc., La Jolla, CA, USA). Statistical significance was determined based on the mean values ± SD from three independent experiments. Multiple group comparisons were conducted using one-way ANOVA with a Tukey post hoc test to compare multiple groups. Two‐way ANOVA with Tukey’s post hoc test was used to compare multiple groups with two categorical variables. ^*^P < 0.05, ^**^P < 0.01, and ^***^P < 0.001 compared with the indicated groups.

## Results

### MCD-induced liver damage is attenuated by ALI in a mouse model of MASH

**Fig. 1:**
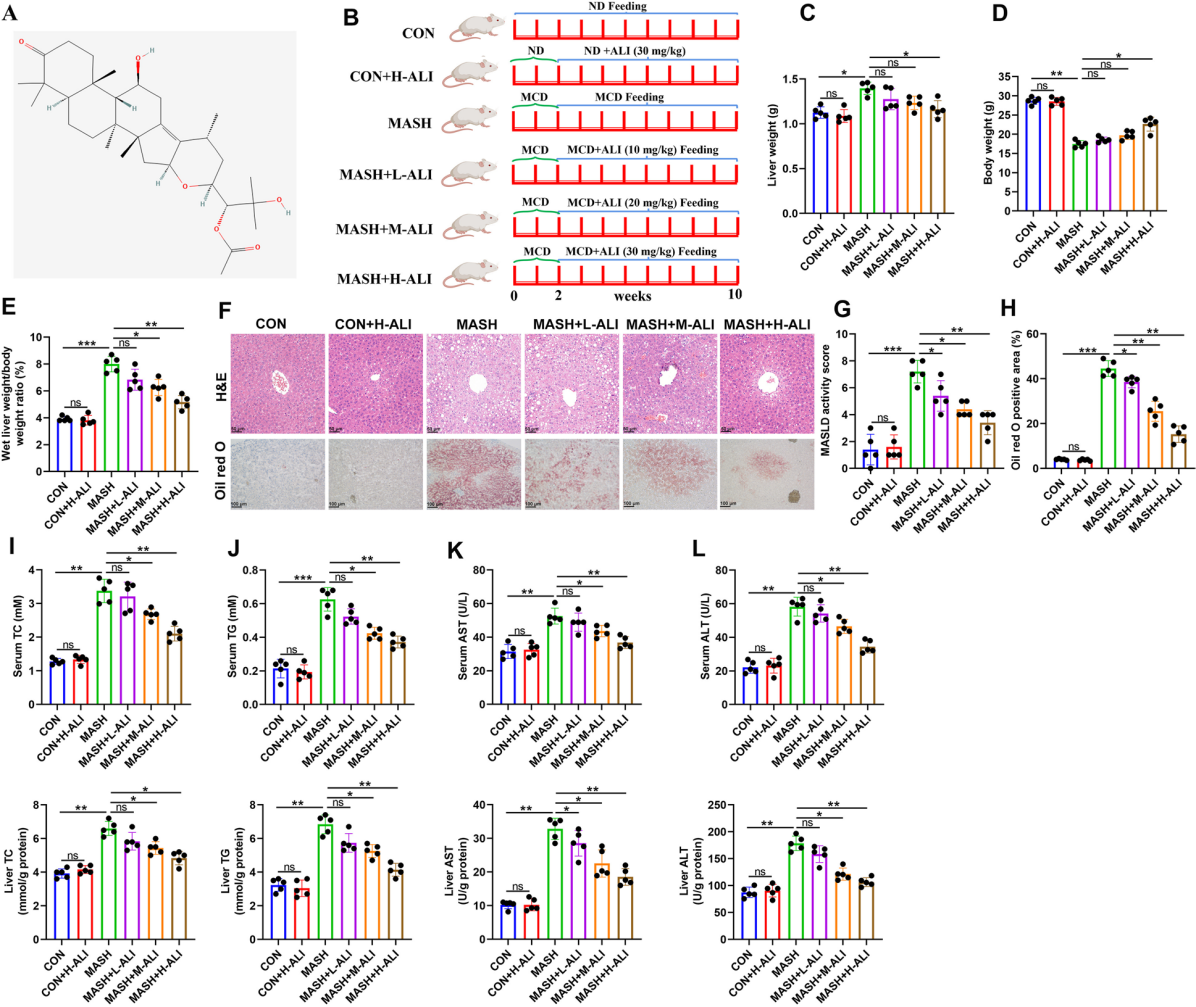
Alisol F 24-acetate (ALI) attenuates methionine–choline-deficient (MCD) diet-induced liver damage in a mouse model of Metabolic Dysfunction-Associated Steatohepatitis (MASH). **A** Chemical structure of ALI. **B** Experimental design for determining the effects of ALI on MCD-treated mice. **C** Effect of ALI on body weight changes in each group of mice. **D** Liver weight of mice at the end of the experiment (week 10). **E** Liver/body weight ratio of rats at the end of the experiment (week 10). **F** Representative images showing **H**&**E** staining (Scale bar, 50 μm) and oil red O staining (Scale bar, 100 μm) for each treatment group. **G** Quantification of the metabolic dysfunction-associated steatotic liver disease (MASLD) activity score. **H** Quantification of oil red O staining. **I**–**L** Circulating levels and hepatic levels of triglyceride (TG), cholesterol (TC), aspartate aminotransferase (AST), and alanine aminotransferase (ALT). Data are mean ± SD (n = 5/group). Statistical significance was determined using one-way ANOVA with a Tukey post hoc test to compare multiple groups. ^*^P < 0.05, ^**^P < 0.01, ^***^P < 0.001 compared with the indicated groups

ALI shares structural similarities with other triterpenoids, such as Alisol A 24-acetate, which is also known to influence liver disease [26]. These compounds are characterized by multi-ring structures and functional groups (Fig. 1A). The structure of triterpenoids enables interactions with biological pathways related to lipid metabolism and inflammation. Their ability to regulate lipid synthesis and reduce inflammation makes them potential candidates for managing liver conditions such as MASH. Therefore, we examined the effects of ALI on the experimental groups of mice treated with various concentrations of ALI (0, 10, 20, or 30 mg/kg) over 10 weeks (Fig. 1B). Our results indicate that ALI could reduce liver weight and increase the body weight of the mice with MCD-induced MASH in a dose-dependent manner, with a high dose (30 mg/kg) having the greatest effect (Fig. 1C–E). Liver sections from MASH mice have a paler appearance when stained with H&E, which may indicate increased hepatocyte ballooning and steatosis, which are hallmarks of MASH (Fig. 1F). The treated sections showed levels of staining closer to the control, suggesting dose-dependent reduced inflammation and improved liver architecture, and these effects are reflected in the MASLD activity score (Fig. 1G). Increased staining in oil red O-stained sections indicates the accumulation of lipids (Fig. 1H). The absence of staining in the control sections suggests minimal fat deposition. The dose-dependent improvement observed with ALI reflects its ability to reduce lipid accumulation. The other parameters we measured, including serum and liver TC, TG, AST, and ALT, were consistent with our other observations (Fig. 1I–L). Overall, our findings demonstrate that ALI can attenuate MCD-induced liver damage in mice.

### ALI reduces hepatic inflammation and fibrosis in MCD-induced liver disease

**Fig. 2:**
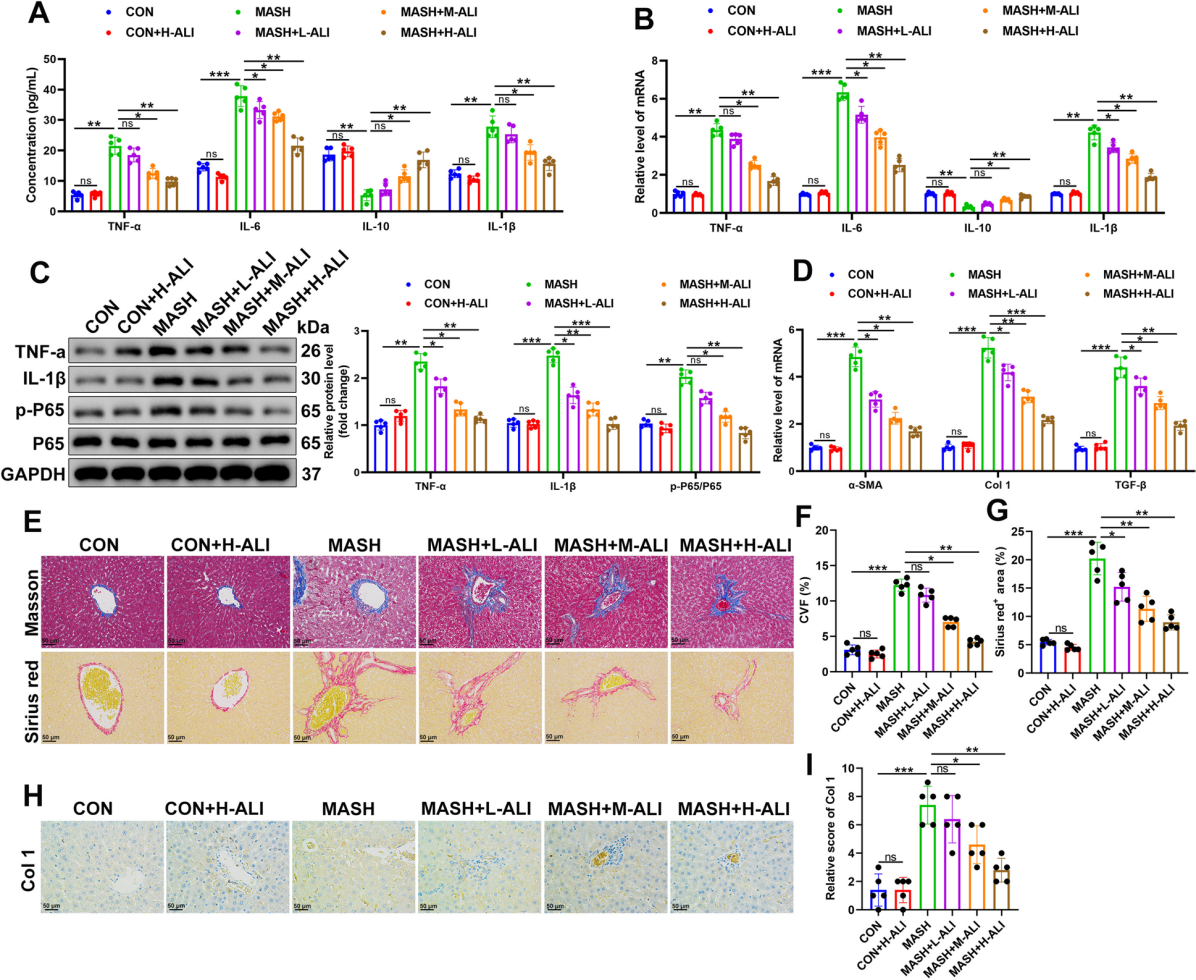
ALI inhibits hepatic inflammation and fibrosis in methionine–choline-deficient (MCD) mice. **A** The expression of TNF-α, IL-6, IL-10, and IL-1β was measured by ELISA in liver tissue. **B** The mRNA levels of TNF-α, IL-6, IL-10, and IL-1β in mouse liver samples were determined by qRT-PCR. **C** Protein levels of p-P65/P65, TNF-a, and IL-1β were detected by western blotting and analyzed using densitometric statistics. **D** mRNA levels of α-Sma, TGF-β, and Col 1 in the liver were measured by quantitative PCR. **E** Representative images of Masson staining and Sirius red staining. **F** Quantification of the collagen volume fraction (CVF). **G** Quantification of Sirius red staining. **H** Immunohistochemical staining was performed to detect the expression of Col 1 in liver tissues. **I** Col 1 staining intensity score. Data are mean ± SD (*n* = 5/group). Statistical significance was determined using one-way ANOVA with a Tukey post hoc test to compare multiple groups. Scale bar, 50 μm. ^*^P < 0.05, ^**^P < 0.01, ^***^P < 0.001 compared with the indicated groups

To determine the extent of inflammation in MCD-induced liver disease, we measured the expression of inflammatory factors in liver tissues treated with various concentrations of ALI (0, 10, 20, or 30 mg/kg) over 10 weeks. The expression of TNF-α, IL-6, IL-1β, and IL-10 in mouse liver tissue was measured by ELISA and qRT-PCR (Fig. 2A, B). Levels of the pro-inflammatory cytokines TNF-α, IL-6, and IL-1β expression were increased by MCD-induced liver damage, whereas those of the anti-inflammatory cytokine IL-10 were reduced, indicating elevated levels of inflammation. However, treatment with ALI reduced the expression of TNF-α, IL-6, and IL-1β and increased the expression of IL-10. This indicates that ALI was able to ameliorate the effects of MCD on the liver, and this effect was dose-dependent. The protein levels of TNF-α and IL-1β were elevated with MCD-induced liver damage and the phosphorylation of P65 was increased (Fig. 2C). Phosphorylation of p65 enhances its ability to translocate to the nucleus and bind DNA, leading to the transcription of pro-inflammatory genes such as TNF-α and IL-1β. In the MASH model, mice treated with ALI exhibited reduced levels of TNF-α, IL-1β, and phosphorylated P65. We also measured the mRNA levels of α-Sma, Col 1, and TGF-β, proteins used to measure fibrosis and collagen deposition. Levels of all three proteins were elevated after liver damage; however, ALI effectively reduced the expression dose-dependently (Fig. 2D). Masson and Sirius red staining revealed the extent of fibrosis and collagen deposition (Fig. 2E–G). The collagen volume fraction was higher in the MASH model, but treatment with ALI could alleviate this. Immunohistological staining of tissue samples to detect the location of Col 1 was able to demonstrate the intensity of collagen deposition within damaged liver tissue (Fig. 2H, I). Collectively, these results indicate a high level of inflammation and fibrosis in MCD-damaged liver tissue, which is attenuated by treatment with ALI.

### ALI mitigates M1/M2 macrophage polarization

**Fig. 3:**
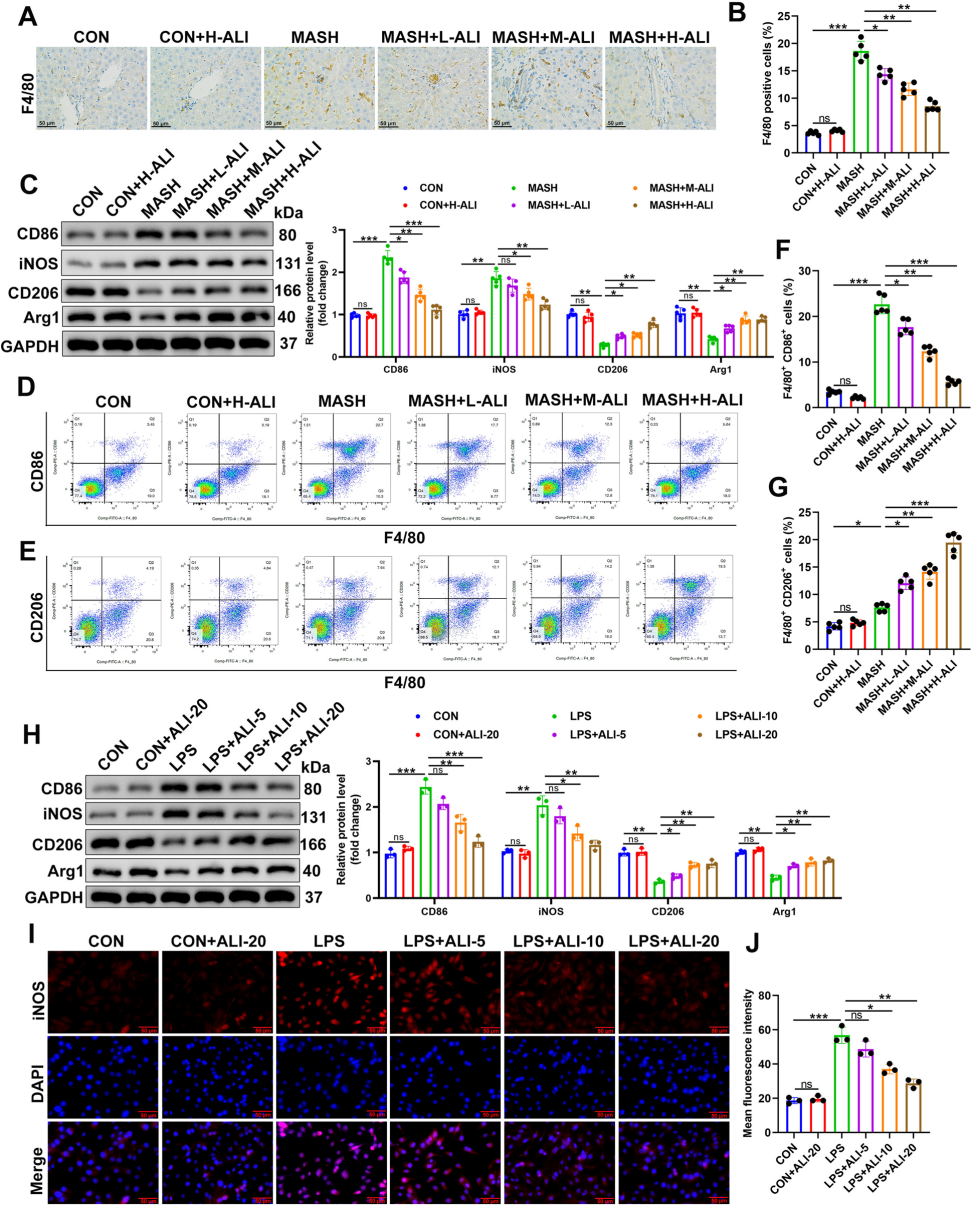
ALI alleviates M1/M2 macrophage polarization. **A**, **B** Representative F4/80 immunohistochemistry in each treatment group, the number of F4/80 positive macrophages was calculated (*n* = 5/group). **C** The expression levels of M1 (CD86, iNOS) and M2 (CD206, Arg1) macrophage markers were measured using western blotting (*n* = 5/group). **D**–**G** Representative flow cytometry plots showing macrophage distribution by using CD86 (**D**) and CD206 (**E**), and quantification of M2 and M1 in the liver (**F**, **G**) (n = 5/group). RAW264.7 cells stimulated by using LPS and ALI, **H** Protein expression of CD86, iNOS, CD206, and Arg1 assayed by western blotting and quantitative analysis (*n* = 3). **I**, **J** iNOS levels were visualized by immunofluorescence, and the number of iNOS-positive cells was calculated (*n* = 3). Data are mean ± SD. Statistical significance was determined using one-way ANOVA with a Tukey post hoc test to compare multiple groups. Scale bar, 50 μm. ^*^P < 0.05, ^**^P < 0.01, ^***^P < 0.001 compared with the indicated groups

Macrophage polarization plays a crucial role in the development of liver inflammation and fibrosis [7]. We next determined whether the increase in inflammation observed in MCD-damaged liver tissue and its subsequent alleviation by ALI were associated with macrophage polarization. F4/80, a member of the EGF-TM7 family, can be used to detect mature macrophages [27]. Immunohistochemistry indicated higher levels of F4/80-positive cells in mice with MCD-damaged liver tissue compared to the control (Fig. 3A, B). However, treatment with ALI lowered the number of F4/80-positive cells in damaged liver tissue dose-dependently. To investigate this in greater depth, we measured the levels of M1 (CD86, iNOS) and M2 (CD206, Arg1) macrophage markers by western blotting (Fig. 3C). Levels of M1 macrophage markers were increased in mice with MCD-induced liver damage, whereas treatment with ALI reduced these levels. In contrast, levels of M2 macrophage markers were reduced by MCD and increased by ALI dose-dependently. This demonstrates that liver tissue in the MASH mouse model contained a greater number of M1 macrophages and fewer M2 macrophages. However, treatment with ALI was able to reverse this trend. Flow cytometry plots of F4/80-positive cells using the M1 marker CD86 and the M2 marker CD206 substantiated these findings (Fig. 3D–G). Elevated numbers of M1 macrophages are found in the MASH model with lower numbers of M2 macrophages (Fig. 3D, E). However, ALI can mitigate the effects of MASH dose-dependently (Fig. 3F, G). Similar results were obtained with RAW264.7 cells (Fig. 3H–J) and BMDMs (Fig. S1A-C) stimulated with LPS. Protein expression of the M1 macrophage markers CD86 and iNOS and the M2 macrophage markers CD206 and Arg1 was measured by western blotting and quantitative analysis. The results demonstrated that there was a higher level of M1 macrophage and a lower number of M2 macrophage in LPS-stimulated cells (Fig. 3H; Fig. S1A). However, ALI could mitigate M1/M2 macrophage polarization in a dose-dependent manner. The effects were also visualized by immunofluorescence using iNOS (Fig. 3I; Fig. S1B), and the number of iNOS-positive cells was calculated (Fig. 3J; Fig. S1C). The iNOS enzyme is a hallmark of pro-inflammatory M1 macrophages. These macrophages produce nitric oxide via iNOS in MASH, which amplifies the inflammatory response [28]. The immunofluorescence images confirmed that ALI alleviated the effects of LPS-stimulated M1/M2 macrophage polarization. Taken together, our findings indicate that pro-inflammatory M1 macrophages play a dominant role in MASH progression. Importantly, ALI demonstrates the ability to mitigate these effects.

### ALI inhibits macrophage pyroptosis

**Fig. 4:**
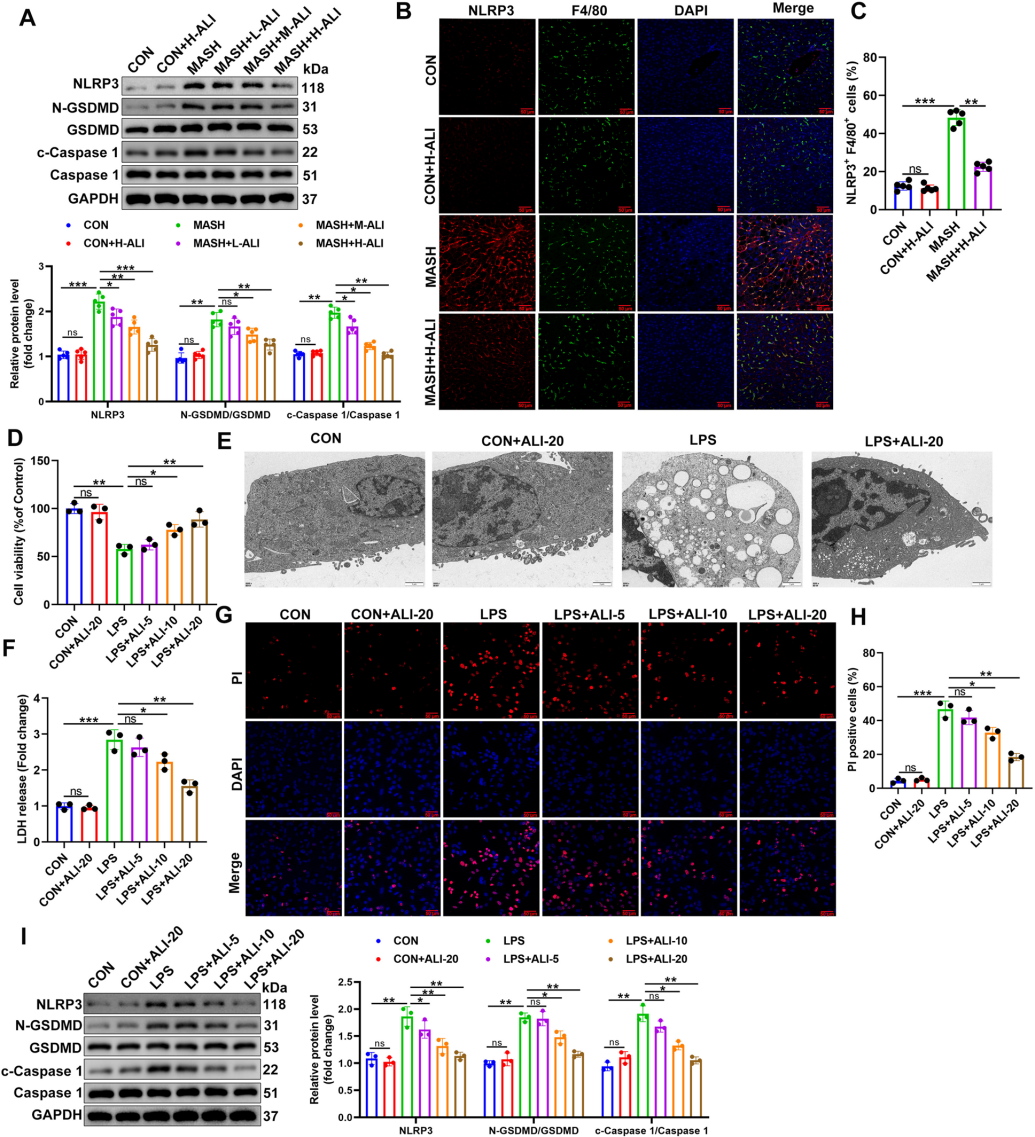
ALI inhibits macrophage pyroptosis. **A** Protein levels and relative intensity ratios of NLRP3, c-Caspase-1/Caspase-1, and N-GSDMD/GSDMD normalized to GAPDH in mouse liver tissues (n = 5/group). **B** Immunofluorescent staining of NLRP3 (Red) and F4/80 (Green); Blue, DAPI (n = 5/group). **C** The percentage of NLRP3-F4/80 positive cells was calculated. **D** CCK-8 was used to evaluate the cell viability of RAW264.7 cells exposed to LPS and ALI for 48 h (*n* = 3). **E** The features of pyroptosis in RAW264.7 cells were detected by TEM (Scale bar, 1 µm). **F** Levels of lactate dehydrogenase (LDH) detected in cells (*n* = 3). **G**, **H** PI staining was used to evaluate cell viability (*n* = 3). **I** Protein expression of NLRP3, c-Caspase-1/Caspase-1, N-GSDMD/GSDMD measured in RAW264.7 cells by western blotting with GAPDH as a loading control (*n* = 3). Data are mean ± SD. Statistical significance was determined using one-way ANOVA with a Tukey post hoc test to compare multiple groups. Scale bar, 50 μm. ^*^P < 0.05, ^**^P < 0.01, ^***^P < 0.001 compared with the indicated groups

Macrophage pyroptosis, a form of inflammatory programmed cell death, plays a crucial role in MASH and other liver diseases, where pyroptosis activation plays a critical role in M1 macrophage polarization [29, 30]. In this study, we measured levels of NLRP3, c-Caspase-1/Caspase-1, and N-GSDMD/GSDMD in mouse liver tissue (Fig. 4A). No change occurred between the control and the mice administered the highest level of ALI (30 mg/kg), indicating that ALI did not contribute to pyroptosis. However, the MASH mouse model resulted in the highest levels of NLRP3, c-Caspase-1/Caspase-1, and N-GSDMD/GSDMD, indicating that pyroptosis is the most severe in this group. Treating the MASH mouse model with increasing concentrations of ALI (10, 20, 30 mg/kg) lowered the levels of NLRP3, c-Caspase-1/Caspase-1, and N-GSDMD/GSDMD dose-dependently. These results were confirmed by the immunofluorescence staining of NLRP3-F4/80 positive cells (Fig. 4B). The percentage of NLRP3-F4/80 positive cells was the highest in the MASH model. However, treatment with the highest dose of ALI lowered the level of NLRP3-F4/80 positive cells by approximately 50% (Fig. 4C). A similar pattern occurred in RAW264.7 cells (Fig. 4D) and BMDMs (Fig. S2A) exposed to LPS and ALI for 48 h. Cell viability was the lowest in cells exposed to LPS. However, ALI was able to improve cell viability dose-dependently. TEM was used to determine the RAW264.7 cell morphology. The results showed that LPS-treated cells had pyroptosis features, including cell swelling, low density of cytosol, and pore formation on the cell membrane. ALI treatment mitigated the pyroptotic characteristics induced by LPS (Fig. 4E). Cell membrane integrity was determined using an LDH release assay (Fig. 4F; Fig. S2B). The LDH release was highest in the LPS-challenged cells. However, ALI was able to lower the amount of LDH released. These results were validated and visualized by using a PI assay (Fig. 4G, H; Fig. S2C, 2D). Finally, levels of NLRP3, c-Caspase-1/Caspase-1, and N-GSDMD/GSDMD were measured in RAW264.7 (Fig. 4I) and BMDMs (Fig. S2E) by western blotting. The results confirmed that ALI was able to inhibit macrophage pyroptosis, and these effects were dose-dependent.

### NLRP3-mediated macrophage pyroptosis is inhibited by ALI

**Fig. 5:**
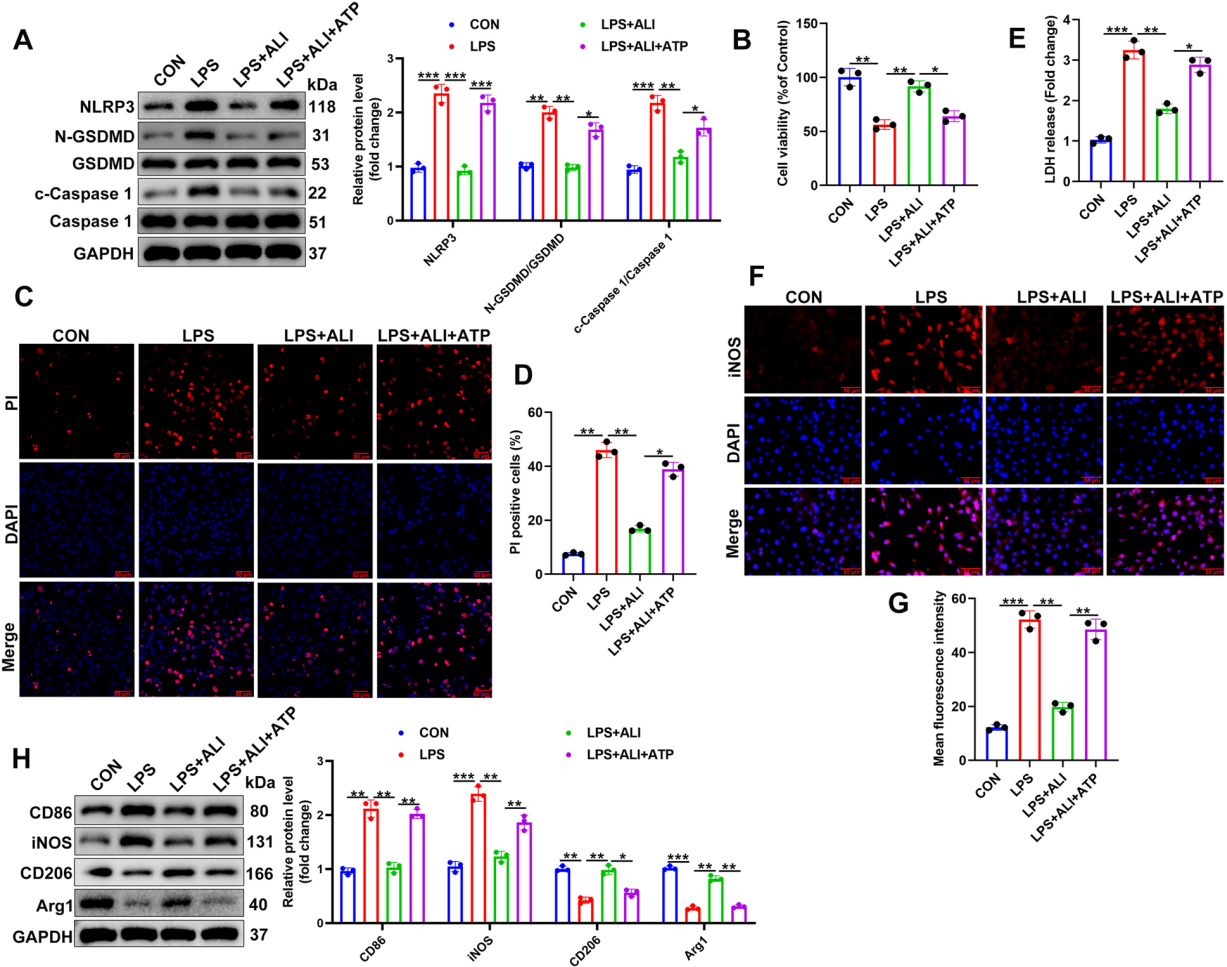
ALI inhibits NLRP3-mediated macrophage pyroptosis. Lipopolysaccharide (LPS, 250 ng/ml) stimulation of RAW264.7 cells was accompanied by ALI and ATP (5 mM) treatment for 24 h. **A** The effects of ALI and ATP on pyroptosis were assessed using western blotting. **B**–**D** CCK-8 and Hochest/PI staining are used to evaluate the cell viability in different groups. **E** Levels of LDH in the cell culture supernatant of different treatment groups. **F**, **G** iNOS levels were detected by immunofluorescence, and the number of iNOS-positive cells was calculated. **H** Protein expression of CD86, iNOS, CD206, and Arg1 assayed by western blotting and quantitative analysis. Data are mean ± SD (*n* = 3). Statistical significance was determined using one-way ANOVA with a Tukey post hoc test to compare multiple groups. Scale bar, 50 μm. ^*^P < 0.05, ^**^P < 0.01, ^***^P < 0.001 compared with the indicated groups

Elevated expression of NLRP3 was mainly observed in MDMs in MASH patients with fibrosis, indicating a potential link between macrophages, NLRP3, and liver disease progression. To gain deeper insight into how ALI prevents macrophage pyroptosis in vitro, we used ATP (specific NLRP3 inflammasome activator) co-treatment with ALI to explore the effect of ALI on NLRP3 inflammasome activation. Western blotting was used to measure levels of NLRP3, c-Caspase-1/Caspase-1, and N-GSDMD/GSDMD in LPS-stimulated RAW264.7 macrophage cells treated with ALI for 24 h (Fig. 5A). Levels of NLRP3, c-Caspase-1/Caspase-1, and N-GSDMD/GSDMD demonstrate that ALI prevents macrophage pyroptosis. However, the addition of ATP can reverse the beneficial effects of ALI. ATP can override the protective effects of certain drugs by triggering oxidative stress and inflammasome assembly [31]. Our results demonstrate that the addition of ATP counteracts the benefits of administering ALI. Measurements of cell viability by Hoechst/PI staining and CCK-8 produced similar results (Fig. 5B–D). The loss of cell viability induced by LPS is restored by ALI to some extent. However, ATP intensifies the effect of LPS, leading to a higher loss of cell viability despite the presence of ALI. Similarly, LDH released into the cell culture of different treatment groups indicates that cell membrane integrity is greater in the LPS-injured cells treated with ALI than in those counter-challenged with ATP (Fig. 5E). Immunofluorescence demonstrated that the addition of ATP also increased the level of iNOS-positive LPS-stimulated macrophage cells treated with ALI (Fig. 5F, G). Elevated levels of CD86 and iNOS and lower levels of CD206 and Arg1 were found in cells challenged by both LPS and ATP, even when treated with ALI, indicating a shift toward M1 macrophage polarization (Fig. 5H). The above results suggest that ALI can attenuate pyroptosis by reducing NLRP3 inflammasome activation in vitro*.*

### ALI acts as an exogenous antioxidant in LPS-stimulated macrophages and the MASH mouse model

**Fig. 6:**
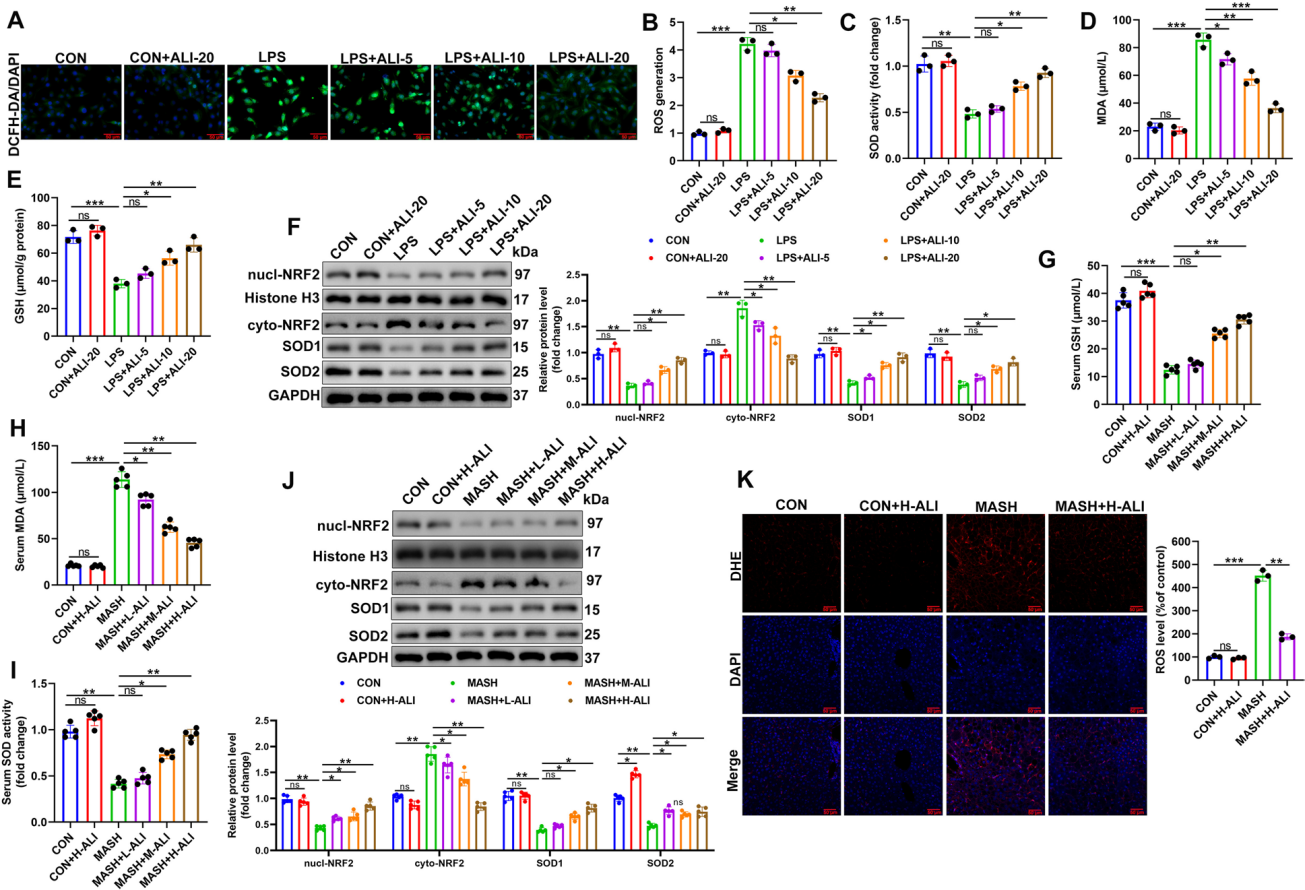
ALI protects LPS-treated macrophages and a mouse MASH model from oxidative stress. **A** and **B** ROS generation was analyzed and quantified by 2′,7′-dichlorofluorescein diacetate (DCFH-DA) via fluorescence microscopy (*n* = 3). **C**–**E** SOD, GSH, and MDA levels in RAW264.7 cells (*n* = 3). **F** Representative western blot of cyto-NRF2, Nucl-NRF2, SOD1, and SOD2 proteins (*n* = 3). **G**–**I** SOD, GSH, and MDA levels in serum (*n* = 3). **J** Representative western blot of cyto-NRF2, Nucl-NRF2, SOD1, and SOD2 proteins in liver tissue (*n* = 5/group). **K** Representative fluorescence images of liver tissue stained with dihydroethidium (DHE) and quantification of DHE staining (*n* = 5/group). Data are mean ± SD. Statistical significance was determined using one-way ANOVA with a Tukey post hoc test to compare multiple groups. Scale bar, 50 μm. ^*^P < 0.05, ^**^P < 0.01, ^***^P < 0.001 compared with the indicated groups

Increasing evidence suggests that cellular oxidative damage is an important trigger for NLRP3-mediated activation of focal death, which is mediated by KEAP1/NRF2 signaling [32]. We further investigated the antioxidant properties of ALI in LPS-stimulated macrophages and mice with MCD-induced MASH. The generation of ROS was measured by fluorescence in LPS-stimulated RAW264.7 macrophage cells stained with DCFH-DA (Fig. 6A, B). ALI was able to diminish ROS generation in these macrophages dose-dependently. Similar results were observed for cellular protein levels of SOD, MDA, and GSH (Fig. 6C–E; Fig. S3A-C). High levels of SOD and GSH with lower levels of MDA indicate an enhancement of cellular defense against oxidative stress, possibly due to the activation of the NRF2 pathway. However, ALI demonstrates antioxidant properties by reversing these changes dose-dependently. To explore the activity of NRF2 further, we measured the levels of cyto-NRF2, nucl-NRF2, SOD1, and SOD2 in RAW264.7 macrophage cells by western blotting (Fig. 6F). In response to LPS, the macrophage contained high levels of cyto-NRF2, low levels of nucl-NRF2, and low levels of SOD1 and SOD2. This indicates impaired NRF2 nuclear translocation and weakened antioxidant defense, possibly due to excessive KEAP1-mediated degradation or disrupted signaling. Treatment with ALI was able to stimulate the weakened antioxidant defense by increasing NRF2 nuclear translocation and the activities of SOD1 and SOD2. In BMDMs, ALI similarly reversed LPS-induced NRF2 cytoplasmic translocation, leading to increased NRF2 nuclear translocation (Fig. S3D). We replicated these experiments in a mouse model of MASH. As in the macrophage cells, we found that levels of SOD, GSH, and MDA in mouse serum indicated increased oxidative stress in the MCD-treated mice, which was alleviated by ALI dose-dependently (Fig. 6G–I). Western blotting to measure levels of cyto-NRF2, Nucl-NRF2, SOD1, and SOD2 proteins also indicated impaired NRF2 nuclear translocation and weakened antioxidant defense in the liver tissue of MCD-treated mice (Fig. 6J). ALI was found to restore the antioxidant defense in the mouse livers to some extent. We found that the DHE-stained liver tissue of the MCD-treated mice contained the highest levels of ROS (Fig. 6K). However, ALI was able to stimulate an antioxidant response, lowering the level of ROS. Overall, our results suggest that NRF2 activation impaired by MASH can be restored by ALI to lower the generation of ROS.

### ALI targets the regulation of KEAP1/NRF2 signaling in MASH

**Fig. 7:**
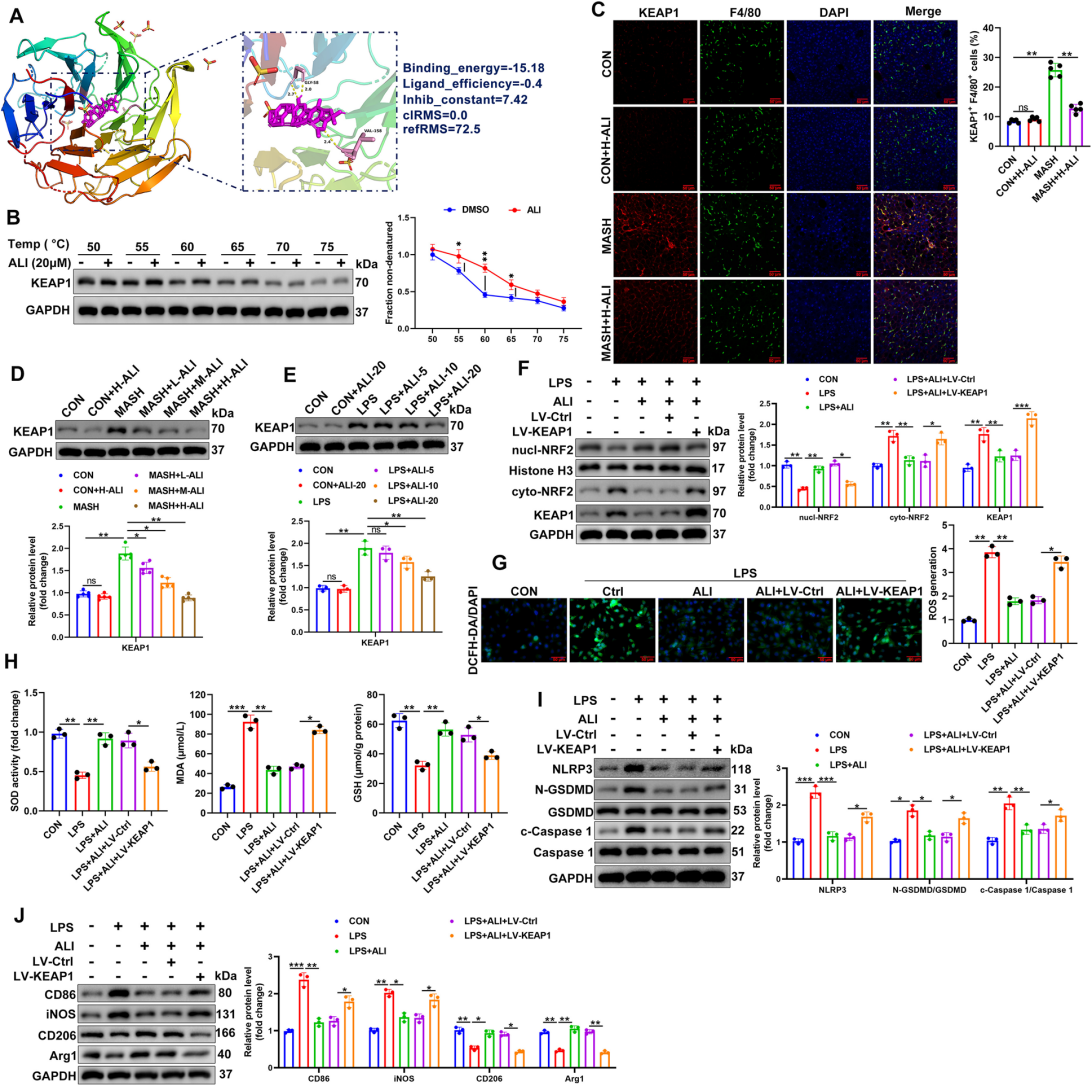
Alisol F 24-acetate (ALI) targets KEAP1/NRF2 signaling in MASH. **A** ALI molecular docking data were analyzed by using Autodock software, Binding energy =  − 15.18, Ligand efficiency =  − 0.4, Inhib constant = 7.42, clRMS = 0.0, refRMS = 72.5. **B** Cellular thermal shift assay analysis of the binding and quantification of ALI and KEAP1 proteins. **C** The immunoreactivities of F4/80 and KEAP1 in liver tissues were detected by immunofluorescence staining: Red, KEAP1; Green, F4/80; Blue, DAPI (n = 5/group). **D** Western blotting to detect KEAP1 protein levels in mouse liver tissues (*n* = 5/group). **E** RAW264.7 cells stimulated using LPS and ALI, the protein expression of Keap1 was assayed by western blot and quantitative analysis. RAW264.7 cells were transduced with OE-Ctrl or OE-*Keap1* for 48 h, followed by LPS and ALI treatment (*n* = 3). **F** Representative western blot of cyto-NRF2, Nucl-NRF2, and KEAP1 proteins (*n* = 3). **G** The analysis of ROS generation through fluorescence microscopy with DCFH-DA (**F**) and quantification of ROS (**G**) (*n* = 3). **H** SOD, GSH, and MDA levels in RAW264.7 cells (*n* = 3). **I** Protein expression of NLRP3, c-Caspase-1/Caspase-1, N-GSDMD/GSDMD assayed by western blotting (*n* = 3). **J** Protein expression of CD86, iNOS, CD206, and Arg1 assayed by western blotting and quantitative analysis (*n* = 3). Data are mean ± SD. Statistical significance was determined using one-way ANOVA with a Tukey post hoc test to compare multiple groups. Two‐way ANOVA with Tukey’s post hoc test was used to compare multiple groups with two categorical variables. Scale bar, 50 μm. ^*^P < 0.05, ^**^P < 0.01, ^***^P < 0.001 compared with the indicated groups

To determine if ALI influences the activation of NRF2, we investigated KEAP1 signaling in the MASH mouse model and macrophage cells. Molecular docking data suggest that ALI has a strong binding affinity to KEAP1, with a binding energy of − 15.18 kcal/mol, ligand efficiency of− 0.4, and an inhibitory constant (Ki) of 7.42 pM. The low RMSD values (reference RMSD = 72.5) and absence of steric clashes (clash detection = 0.0) further confirm the reliability of the docking pose (Fig. 7A). CETSA results indicate that ALI enhances KEAP1 protein thermostability upon heating from 50 °C to 75 °C (Fig. 7B), implicating KEAP1 as a key target for ALI. Protein analysis of KEAP1 in the MASH mouse model by immunofluorescence and western blotting (Fig. 7C, D) suggests that KEAP1 levels are increased in the MASH model. However, ALI lowers the level of KEAP1. This suggests that ALI may be disrupting KEAP1-mediated degradation of NRF2, allowing for enhanced antioxidant response and reduced oxidative damage. Similar results were found in LPS-stimulated macrophages (Fig. 7E; Fig. S3D). Overall, the values suggest that ALI may enhance NRF2 activation by inhibiting KEAP1, leading to increased antioxidant defense and reduced oxidative stress. RAW264.7 cells were transduced with OE-Ctrl or OE-*Keap1* for 48 h, followed by LPS and ALI treatment. Compared to the control, KEAP1 protein levels are increased by LPS stimulation. However, ALI can modulate the effects of LPS on KEAP1 dose-dependently. Western blotting to measure levels of cyto-NRF2, Nucl-NRF2, and KEAP1 proteins in LPS-stimulated macrophage indicated that impaired NRF2 nuclear translocation is accompanied by higher levels of KEAP1, which are lowered by ALI (Fig. 7F). Immunofluorescence images and ROS, SOD, MDA, and GSH assays show that the overexpression of *Keap1* counteracts the antioxidant actions of ALI in LPS-stimulated cells (Fig. 7G, H). In LPS-stimulated macrophage cells, levels of NLRP3, c-Caspase-1/Caspase-1, and N-GSDMD/GSDMD are increased but lowered when cells are treated with ALI. However, when *Keap1* is overexpressed, levels are increased again (Fig. 7I). This suggests that ALI suppresses pyroptosis by inhibiting the NLRP3 inflammasome pathway, but *Keap1* overexpression reverses this effect. Additionally, the measurement of CD86, iNOS, CD206, and Arg1 in macrophage cells by western blotting indicates that LPS promotes M1 polarization, but ALI can shift macrophage polarization back to M2 (Fig. 7J). However, when *Keap1* is overexpressed, NRF2 activation is blocked, allowing macrophages to remain in a pro-inflammatory state.

### Keap1 overexpression abolishes the anti-MASH effects of ALI

**Fig. 8:**
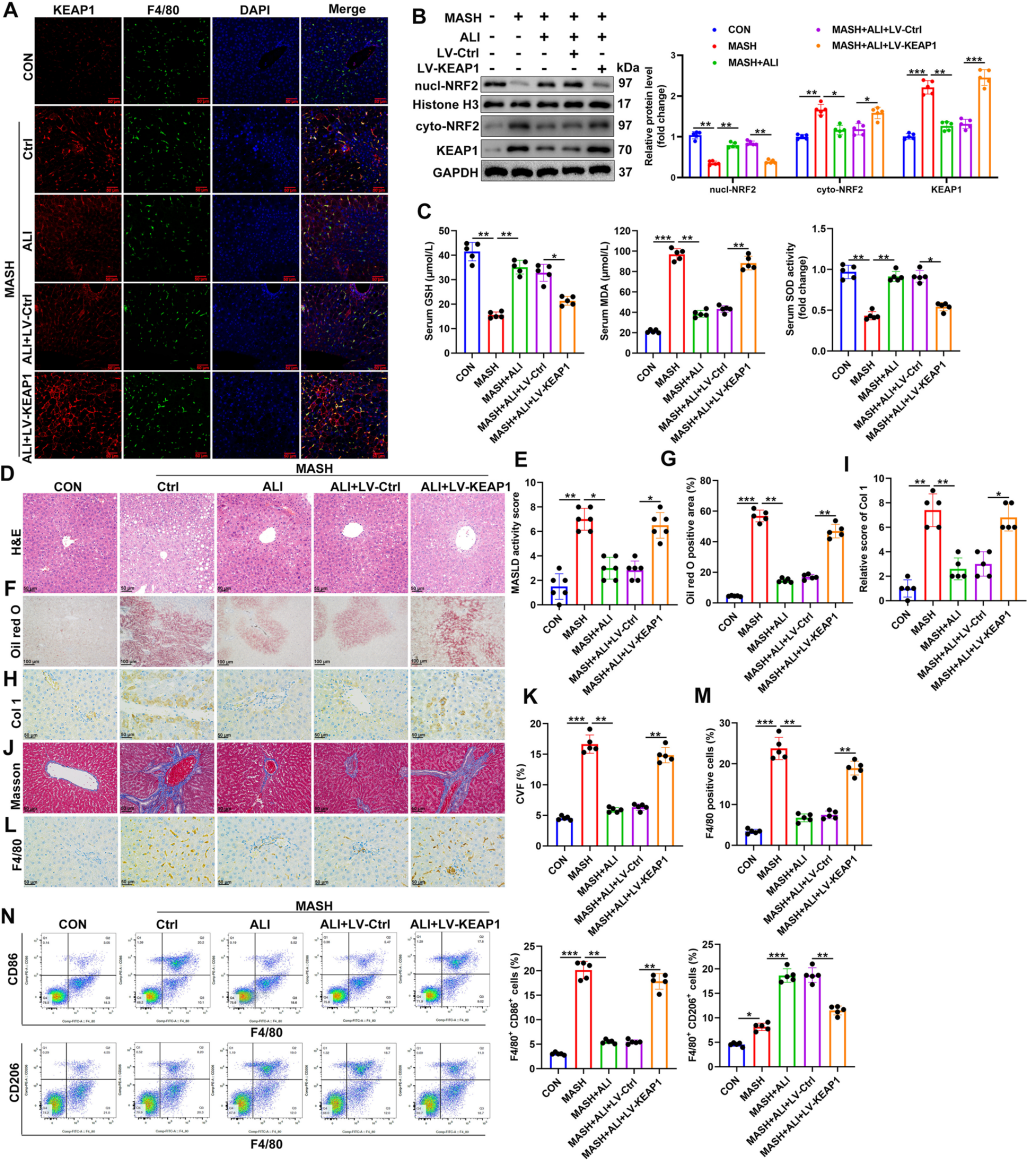
Anti‐MASH effects of ALI are abolished by overexpression of KEAP1. C57BL/6 mice were fed with a normal diet (ND) or a methionine–choline-deficient (MCD) diet. Two groups of MCD-fed mice received tail vein injections of 5 × 10^8^ IU OE-ctrl or OE-*Keap1* packaged lentivirus once every 2 weeks from the beginning of the experiment. They were euthanized after 10 weeks of feeding. **A** Localization of KEAP1 (red) and macrophage marker F4/80 (green) in liver tissues was assessed by immunofluorescence. Nuclei were visualized with DAPI (blue) (Scale bar, 50 μm). **B** Representative western blot of cyto-NRF2, Nucl-NRF2, and KEAP1 proteins. **C** SOD, GSH, and MDA levels in serum. **D** Representative images showing H&E staining for each treatment group; Scale bar, 50 μm. **E** Quantification of the MASLD activity score. **F** Representative images showing oil red O staining for each treatment group (Scale bar, 100 μm). **G** Quantification of the amount of oil red O staining. **H** Immunohistochemical staining was performed to detect the expression of Col 1 in liver tissues (Scale bar, 50 μm). **I** The score of Col 1 staining. **J** Representative images of Masson staining (Scale bar, 50 μm). **K** Quantification of the collagen volume fraction (CVF). **L**, **M** Representative F4/80 immunohistochemistry in each treatment group, the number of F4/80 positive macrophages was calculated (Scale bar, 50 μm). **N** Representative flow cytometry plots showing macrophage distribution by CD86 and CD206, and quantification of M2 and M1 in the liver. Data are mean ± SD (n = 5/group). Statistical significance was determined using one-way ANOVA with a Tukey post hoc test to compare multiple groups. ^*^P < 0.05, ^**^P < 0.01, ^***^P < 0.001 compared with the indicated groups

Finally, we observed the effects of *Keap1* overexpression in the liver and serum of the MASH mouse model. MCD-fed and control mice received a tail vein injection of OE-*Keap1* packaged Lentivirus once every 2 weeks from the beginning of the experiment. We detected the colocalization of F4/80 and KEAP1 in liver tissue (Fig. 8A). The results indicate that the KEAP1 vector has been partially transferred to macrophages. Western blot analysis of cyto-NRF2, Nucl-NRF2, and KEAP1 proteins indicated that impaired NRF2 nuclear translocation and weakened antioxidant defense in the liver tissue of the mice fed an MDC diet were restored by ALI (Fig. 8B). However, the overexpression of *Keap1* reversed this effect. This was confirmed by the serum levels of SOD, GSH, and MDA (Fig. 8C). We found that the overexpression of *Keap1* abolishes the antioxidant effect of ALI. Representative images of H&E-stained liver sections from each treatment group and quantification of the MASLD activity score demonstrate that liver sections from the MASH model have increased levels of steatosis and inflammation (Fig. 8D, E). However, ALI-treated sections had reduced inflammation and improved liver architecture, but the overexpression of *Keap1* abolished these effects. Increased lipid accumulation indicated in oil red O-stained sections was more severe in the MASH model (Fig. 8F, G). An improvement was observed in ALI-treated, but this was counteracted when *Keap1* was overexpressed. Similar findings were obtained with Col1 and Masson staining (Fig. 8H–J). Treatment with ALI alleviated the increased collagen volume fraction (CVF) observed in the MASH model, but *Keap1* overexpression could abolish the effects of ALI (Fig. 8K). Moreover, in the mouse model of MASH, we found that ALI could decrease the level of F4/80 positive cells in the liver expressing CD86 while increasing the level of those expressing CD206 (Fig. 8L–N). However, the overexpression of *Keap1* could counteract the effects of ALI.

**Fig. 9:**
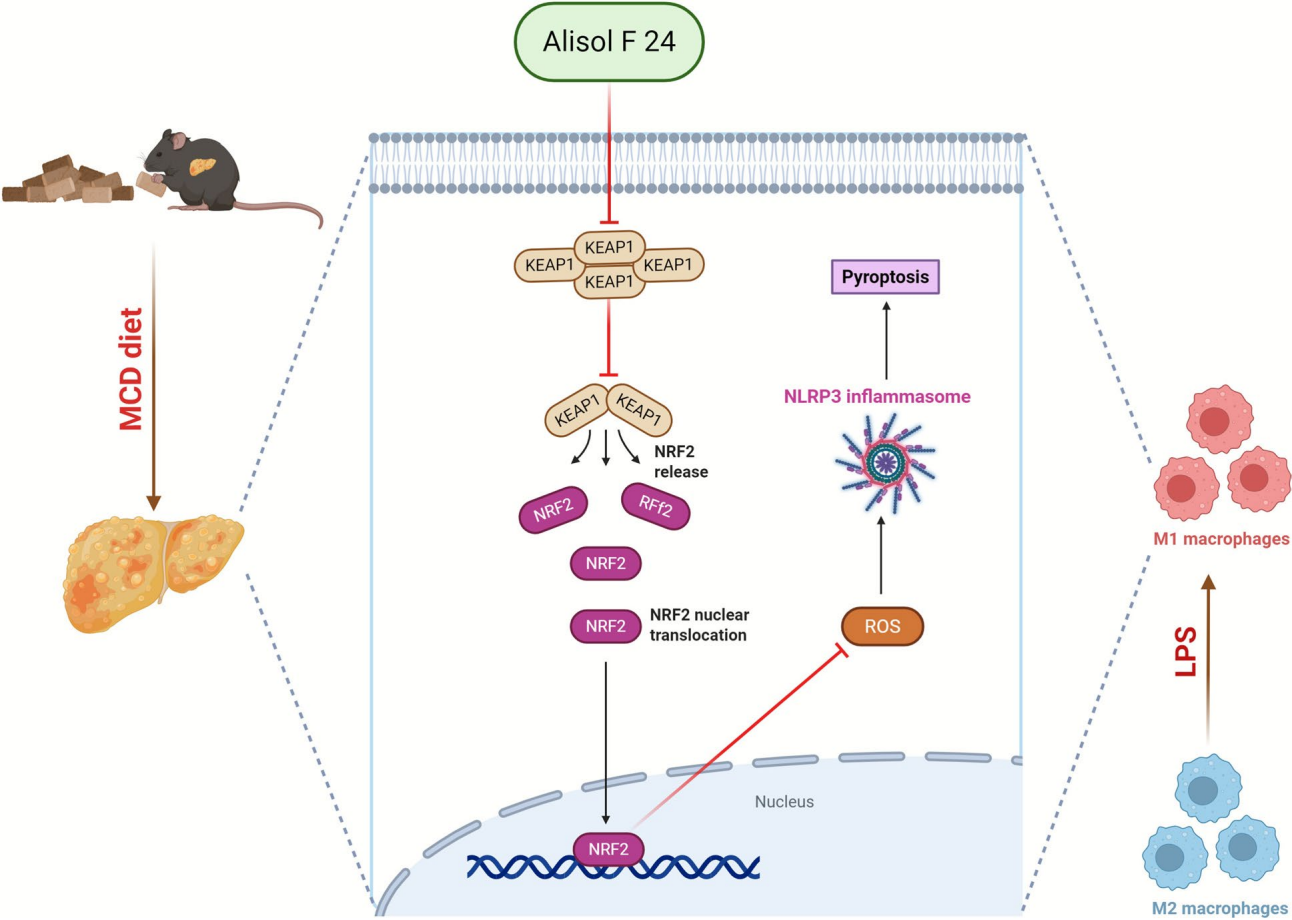
Working model shows that ALI alleviates MASH by inhibiting macrophage polarization via the KEAP1/NRF2-mediated oxidative stress pathway

Cumulatively, these results imply that ALI may be promoting an M2 phenotype, potentially aiding in the resolution of inflammation and fibrosis, whereas the overexpression of *Keap1* may inhibit NRF2 activation, leading to an increase in inflammatory signaling, possibly skewing macrophages back toward the M1 phenotype (Fig. 9).

## Discussion

The MCD diet is a well-characterized experimental model for MASLD, effectively inducing hepatic steatosis that closely mirrors human pathology. Beyond steatosis, this model also elicits a pronounced inflammatory response, making it particularly suitable for evaluating potential therapeutic agents that target inflammatory pathways. Its straightforward induction of fatty liver phenotypes further renders it a practical tool for screening MASLD-specific drug candidates [33, 34]. Clinically, current pharmacotherapies for MASLD primarily consist of hypoglycemic agents, lipid-lowering drugs, and insulin sensitizers. However, the long-term efficacy and safety profiles of these treatments require further validation. Notably, these medications are not specifically designed for MASLD but are instead aimed at managing underlying metabolic conditions such as hyperglycemia or hyperlipidemia [34].

Our study investigated the therapeutic potential of ALI, a triterpene isolated from *Alismatis Rhizoma*, in alleviating the severe symptoms that accompany MASH. We specifically focused on its modulation of the KEAP1/NRF2 signaling pathway in both an MCD-induced MASH mouse model and LPS-stimulated RAW264.7 cells and BMDMs. The results demonstrate that ALI protects against MASH by attenuating hepatic inflammation and fibrosis, regulating macrophage polarization, inhibiting NLRP3-mediated pyroptosis, and acting as an exogenous antioxidant.

MASH is characterized by chronic hepatic inflammation and fibrosis, driven by oxidative stress and immune dysregulation. Our findings align with previous studies showing that targeting oxidative stress pathways can mitigate liver injury [35]. ALI significantly reduced hepatic inflammation and fibrosis in the MCD-induced MASH model, suggesting its potential as a therapeutic agent.

Macrophage polarization plays a crucial role in MASH progression, with M1 macrophages promoting inflammation and M2 macrophages facilitating tissue repair. ALI was found to shift macrophage populations toward an M2 phenotype, reducing the inflammatory burden. This is consistent with previous reports indicating that modulating macrophage polarization can improve MASH outcomes [36].

NLRP3 inflammasome activation contributes to hepatocyte injury and fibrosis in MASH. Our study demonstrates that ALI inhibits NLRP3-mediated macrophage pyroptosis, reducing inflammatory cell death. This aligns with previous research showing that the targeting of NLRP3 can alleviate liver damage in metabolic diseases [22]. The suppression of pyroptosis by ALI may be linked to its ability to enhance NRF2 signaling, which has been shown to counteract inflammasome activation.

An increasing body of evidence suggests that cellular oxidant damage is a significant trigger for the activation of NLRP-mediated pyroptosis, which is modulated by KEAP1/NRF2 signaling [37]. We found that oxidative stress is a key driver of MASH pathogenesis. ALI exhibited strong antioxidant properties in both LPS-stimulated macrophages and the MASH mouse model, reinforcing its potential as a natural antioxidant. Molecular docking studies further support its ability to interact with key oxidative stress regulators. Similar antioxidant effects have been reported for other plant-derived compounds, such as syringic acid, which has been shown to mitigate MASH through multi-target mechanisms [38].

It is well-documented that the KEAP1/NRF2 pathway is a central regulator of oxidative stress and inflammation [39]. Our findings indicate that ALI enhances NRF2 activation, leading to increased expression of antioxidant and cytoprotective genes. However, *Keap1* overexpression abolished the therapeutic effects of ALI against MASH, highlighting the importance of maintaining NRF2 activity. Previous studies have demonstrated that dysregulation of KEAP1/NRF2 signaling contributes to metabolic liver diseases [40]. Interventions targeting this pathway have potential in the management of MASH. In our study, KEAP1 levels are elevated in the MASH mouse model, which could indicate increased oxidative stress and impaired NRF2 activation. KEAP1 is a negative regulator of NRF2, indicating that higher KEAP1 levels could lead to reduced antioxidant defense and heightened inflammation, a hallmark of MASH. However, the fact that ALI lowers KEAP1 levels suggests that ALI may be disrupting KEAP1-mediated degradation of NRF2, allowing for enhanced antioxidant response and reduced oxidative damage. Additionally, the reduction in F4/80-positive cells indicates that ALI may be suppressing macrophage infiltration, which could help mitigate inflammation and fibrosis in the liver. This aligns with studies showing that KEAP1 inhibition can promote NRF2 activation, leading to improved redox balance and reduced inflammatory signaling. If ALI is effectively lowering KEAP1 levels, it may be enhancing NRF2-driven antioxidant defenses, which could be beneficial in MASH treatment.

Our findings suggest that ALI suppresses pyroptosis by inhibiting the NLRP3 inflammasome pathway, but KEAP1 overexpression reverses this effect, leading to increased levels of pyroptosis. Mechanistically, LPS stimulation activates the NLRP3 inflammasome, leading to increased levels of NLRP3, cleaved Caspase-1, and N-GSDMD, which drive pyroptotic cell death. ALI treatment reduces these markers, suggesting it inhibits inflammasome activation, possibly by enhancing NRF2 signaling, which promotes antioxidant defense and suppresses inflammation. KEAP1 overexpression counteracts the effects of ALI by sequestering NRF2, preventing its activation. Since NRF2 plays a role in reducing oxidative stress and inflammation, its inhibition allows NLRP3, cleaved Caspase-1, and N-GSDMD levels to rise again, restoring pyroptosis. This suggests that the protective effects of ALI are NRF2-dependent, and when *Keap1* is overexpressed, it blocks NRF2 activation, allowing pyroptosis to proceed unchecked. To enhance the effects of ALI, targeting KEAP1 degradation or using NRF2 activators might help sustain its anti-inflammatory benefits.

Although our study has several strengths, there are limitations. The primary in vivo evidence was obtained using the MCD diet-induced MASH model. Although this model is widely utilized for its rapid induction of significant steatohepatitis and fibrosis, allowing for clear mechanistic dissection, it does not fully recapitulate the metabolic dysregulation (e.g., insulin resistance, obesity) characteristic of human MASH, which is more faithfully modeled by nutrient-surplus diets such as the Western Diet. Future studies employing metabolic syndrome-associated MASH models will be valuable to confirm the therapeutic potential of Alisol F 24-acetate in a more clinically representative context and to further validate the role of the KEAP1/NRF2 axis in macrophage pyroptosis across different disease etiologies. Moreover, our mechanistic investigation was deliberately focused on macrophages due to their well-established role as central drivers of inflammation and pyroptosis in MASH. However, the KEAP1/NRF2 pathway is a fundamental cytoprotective mechanism that also operates in other hepatic cell types, including hepatocytes and HSCs. Therefore, we cannot exclude the possibility that the observed in vivo benefits of ALI may also involve, to some extent, direct effects on these cells. For instance, NRF2 activation in hepatocytes could ameliorate lipotoxicity and oxidative stress, while its action in HSCs might directly contribute to the anti-fibrotic effect. Investigating how different cell types contribute to the overall therapeutic efficacy of ALI through the KEAP1/NRF2 pathway represents an important direction in our future research. The LPS-stimulated RAW264.7 cells and BMDMs, while an excellent tool for dissecting specific inflammatory and pyroptotic pathways, represents a simplified system that does not fully capture the complex, multi-cellular environment of human MASH. In vivo, macrophages are exposed to a milieu of danger signals beyond LPS, such as free fatty acids, cholesterol crystals, and damage-associated molecular patterns released from stressed or dying hepatocytes. Therefore, future studies using more physiologically relevant setups, such as conditioned media from lipotoxic hepatocytes co-cultured with primary macrophages or investigations in other liver cell types, will be essential to fully validate the role of macrophage KEAP1/NRF2 signaling and to explore potential cell-to-cell communication networks in the anti- MASH effects of ALI. It is also important to address the limitations of our in vivo gene modulation approach. While tail vein injection of lentivirus is a well-established method for hepatic gene delivery, it predominantly transduces hepatocytes rather than offering cell-type-specific targeting. Although immunofluorescence revealed consequent KEAP1 upregulation in liver macrophages, potentially resulting from the altered hepatic microenvironment or paracrine signaling, indirect effects cannot be excluded. Therefore, the specific contribution of KEAP1/NRF2 signaling within macrophages versus other liver cell types to the overall anti- MASH effect of ALI warrants further investigation using more specific genetic tools, such as macrophage-specific conditional knockout or knock-in mouse models.

## Conclusions

In summary, our study provides compelling evidence that ALI exerts hepatoprotective effects in MASH by modulating oxidative stress, macrophage polarization, and inflammasome activation through the KEAP1/NRF2 pathway. These findings contribute to the growing body of research supporting the therapeutic potential of natural compounds in metabolic liver diseases. Future studies should explore the precise molecular interactions of ALI with KEAP1/NRF2 components and assess its clinical applicability.

## Supplementary Information

Supplementary material 1.

## Data Availability

The datasets during the current study are available from the corresponding author on reasonable request.

## Abbreviations

ALI: Alisol F 24-acetate
MASH: Metabolic dysfunction-associated Steatohepatitis
MCD: Methionine–choline-deficient
LPS: Lipopolysaccharide
ROS: Oxygen species
ATP: Adenosine triphosphate
ALT: Alanine aminotransferase
AST: Aspartate aminotransferase
TG: Triglyceride
TC: Cholesterol
SOD: Superoxide dismutase
MDA: Malondialdehyde
GSH: Glutathione
H&E: Hematoxylin and eosin
ELISA: Enzyme-linked immunosorbent assay
LDH: Lactate dehydrogenase
DCFH-DA: Dichlorodi-hydrofluorescein diacetate
PI: Propidium iodide
CVF: Collagen volume fraction
DHE: Dihydroethidium
KEAP1: Kelch-like ECH-associated protein 1
NRF2: Nuclear factor E2-related factor 2
BMDMs: Bone marrow-derived macrophages
