# Supplementary material for: Alisol F 24-acetate attenuated metabolic dysfunction-associated steatohepatitis by targeting the KEAP1/NRF2-mediated macrophage pyroptosis
Source: Chin Med. 2026 Jan 13;21:35. doi: 10.1186/s13020-025-01322-8 (PMC12797777; doi:10.1186/s13020-025-01322-8)
Supplement: Supplementary file 1 — Supplementary material 1. [file 13020_2025_1322_MOESM1_ESM.docx]

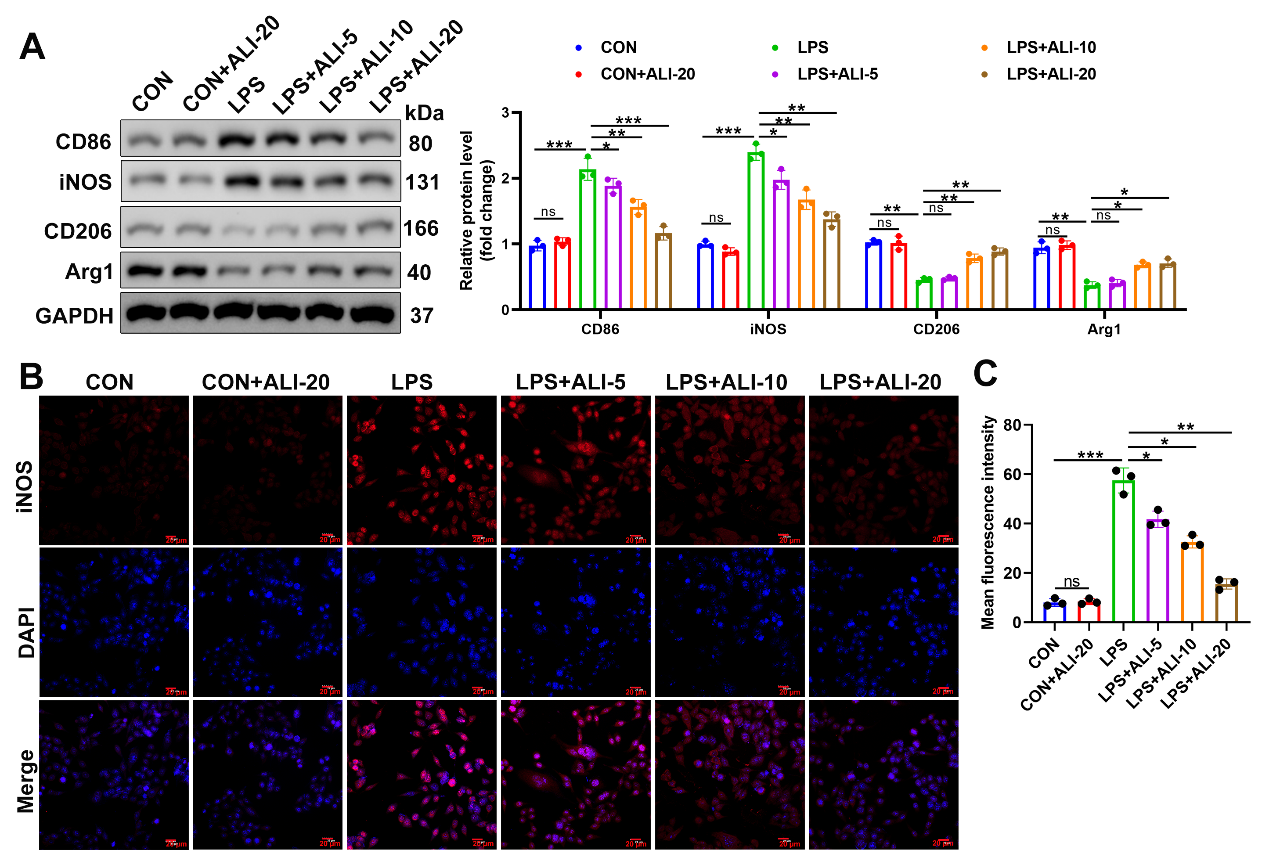


**Supplementary Figure 1. Induction of** **bone marrow-derived macrophage (BMDM) polarization toward M2-like phenotype.** BMDMs were stimulated by using lipopolysaccharide (LPS) and alisol F 24-acetate (ALI). **A.** Protein expression of CD86, iNOS, CD206, and Arg1 was assayed by western blotting and quantitative analysis (n=3). **B, C.** iNOS levels were visualized by immunofluorescence, and the number of iNOS-positive cells was calculated (n=3). Data are mean ± SD. Statistical significance was determined using one-way ANOVA with a Tukey post hoc test to compare multiple groups. Scale bar, 20 μm. ^*^P < 0.05, ^**^P < 0.01, ^***^P < 0.001 compared with the indicated groups.


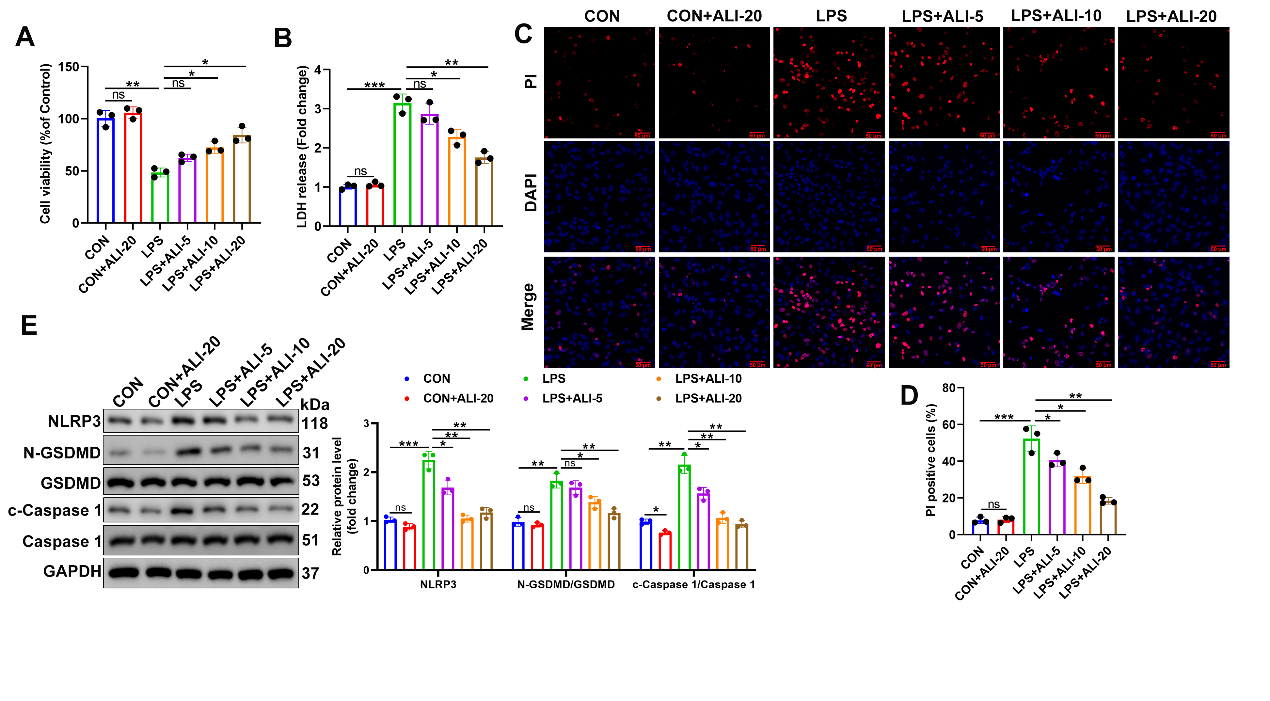


**Supplementary Figure 2. Alisol F 24-acetate (ALI) inhibits pyroptosis in bone marrow-derived macrophage (BMDM).** A. CCK-8 was used to evaluate the cell viability of BMDMs exposed to lipopolysaccharide (LPS) and ALI for 48 h (n=3). **B.** Levels of lactate dehydrogenase (LDH) detected in cells (n=3). **C.** Protein expression of NLRP3, c-Caspase-1/Caspase-1, N-GSDMD/GSDMD measured in BMDMs by western blotting with GAPDH as a loading control (n=3). **D, E.** PI staining was used to evaluate cell viability (n=3). Data are mean ± SD. Statistical significance was determined using one-way ANOVA with a Tukey post hoc test to compare multiple groups. Scale bar, 50 μm. ^*^P < 0.05, ^**^P < 0.01, ^***^P < 0.001 compared with the indicated groups.


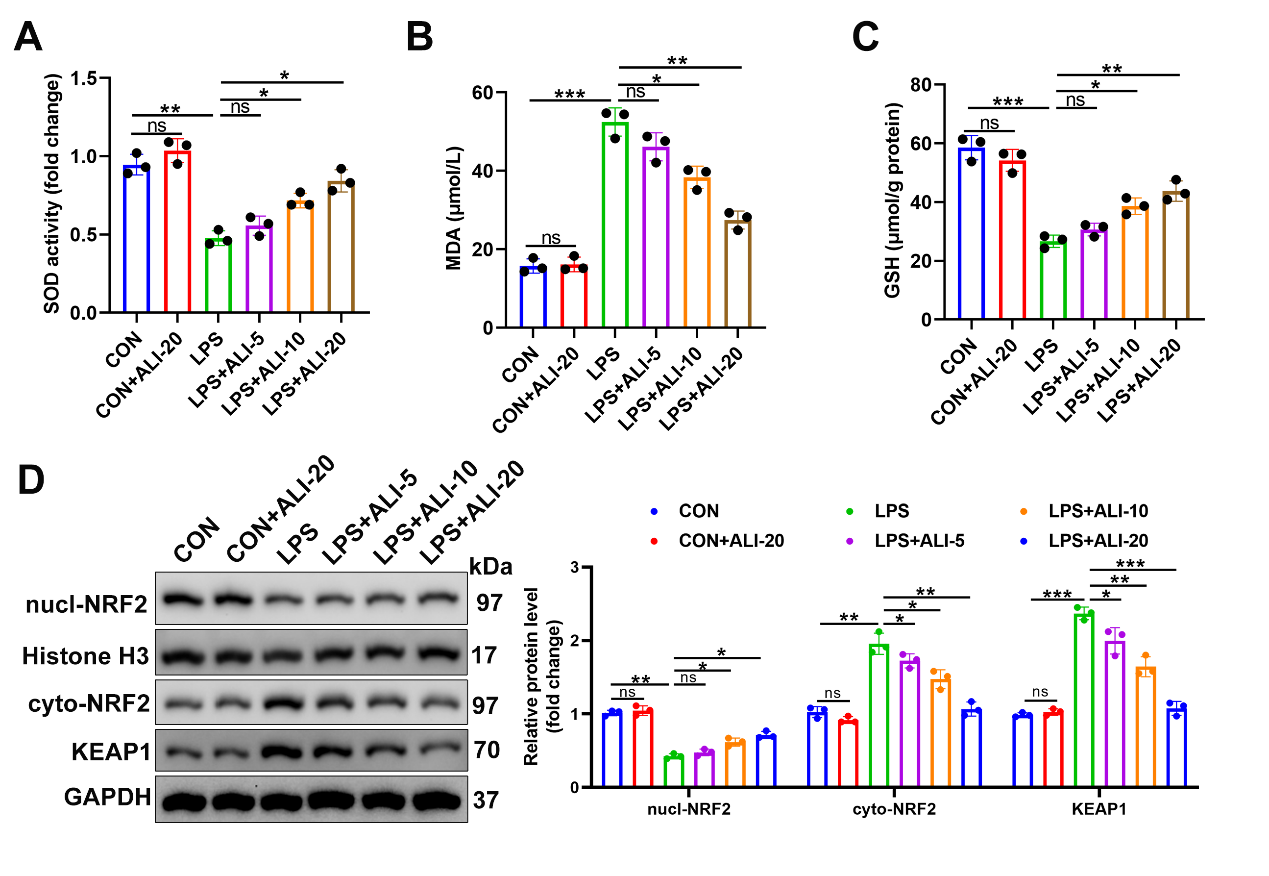


**Supplementary Figure 3. ALI protects LPS-treated** **BMDMs from oxidative stress. A–C.** SOD, GSH, and MDA levels in BMDMs (n=3). **D.** Representative Western blot of cyto-NRF2, Nucl-NRF2, and KEAP1 proteins (n=3). BMDMs were transduced with OE-Ctrl or OE-*Keap1* for 48 h, followed by LPS and ALI treatment (n=3). Data are mean ± SD. Statistical significance was determined using one-way ANOVA with a Tukey post hoc test to compare multiple groups. ^*^P < 0.05, ^**^P < 0.01, ^***^P < 0.001 compared with the indicated groups.
